# Insights into Nano- and Micro-Structured Scaffolds for Advanced Electrochemical Energy Storage

**DOI:** 10.1007/s40820-024-01341-4

**Published:** 2024-02-23

**Authors:** Jiajia Qiu, Yu Duan, Shaoyuan Li, Huaping Zhao, Wenhui Ma, Weidong Shi, Yong Lei

**Affiliations:** 1 Fachgebiet Angewandte Nanophysik, Institut Für Physik and IMN MacroNano, Technische Universität Ilmenau, 98693 Ilmenau, Germany; 2 Key Laboratory of Complex Nonferrous Metal Resources Clean Utilization, Faculty of Metallurgical and Energy Engineering, Kunming University of Science and Technology, Kunming, 650093 People’s Republic of China; 3 School of Chemistry and Chemical Engineering, Jiangsu University, Zhenjiang, 212013 People’s Republic of China; 4 School of Science and Technology, Pu’er University, Pu’er, 665000 People’s Republic of China

**Keywords:** Nano- and micro-structured, Interconnected porous, Scaffolds, Electrode design, Electrochemical energy storage

## Abstract

Adopting a nano- and micro-structuring approach to fully unleashing the genuine potential of electrode active material benefits in-depth understandings and research progress toward higher energy density electrochemical energy storage devices at all technology readiness levels. Due to various challenging issues, especially limited stability, nano- and micro-structured (NMS) electrodes undergo fast electrochemical performance degradation. The emerging NMS scaffold design is a pivotal aspect of many electrodes as it endows them with both robustness and electrochemical performance enhancement, even though it only occupies complementary and facilitating components for the main mechanism. However, extensive efforts are urgently needed toward optimizing the stereoscopic geometrical design of NMS scaffolds to minimize the volume ratio and maximize their functionality to fulfill the ever-increasing dependency and desire for energy power source supplies. This review will aim at highlighting these NMS scaffold design strategies, summarizing their corresponding strengths and challenges, and thereby outlining the potential solutions to resolve these challenges, design principles, and key perspectives for future research in this field. Therefore, this review will be one of the earliest reviews from this viewpoint.

## Highlights

- Recent advances in electrochemical energy storage based on nano- and micro-structured (NMS) scaffolds are summarized and discussed.
- The fundamentals, superiorities, and design principle of NMS scaffolds are outlined.
- Given the present progress, the ongoing challenges and promising perspectives are highlighted.

## Graphical abstract

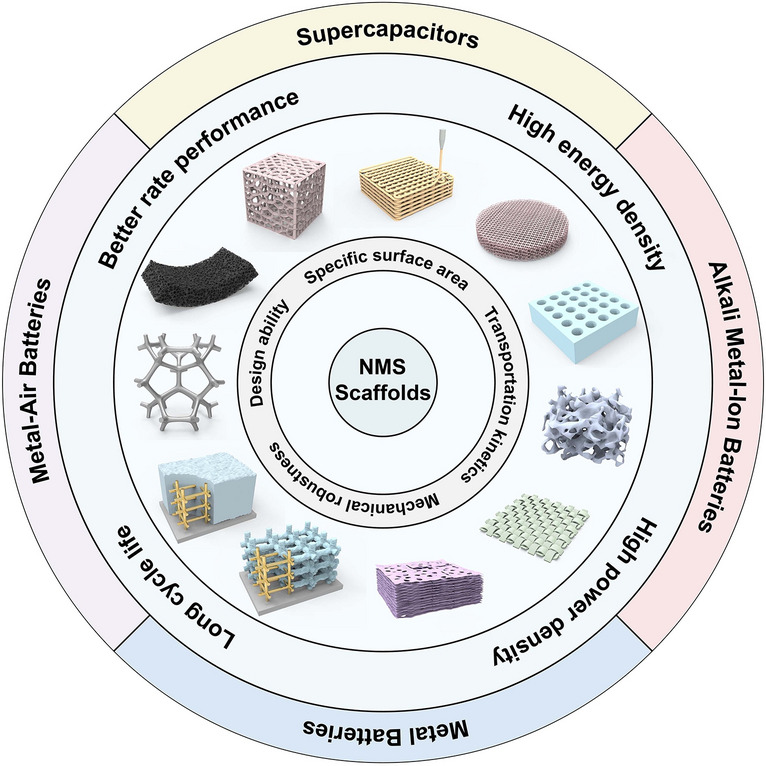

## Introduction

Evolving in leaps and bounds over the past few decades of science and technology, numerous and a variety of high-tech products endow modern life with unprecedented quality than ever before, which has, in turn, ever-accelerated the dependency and desire for higher energy depletion eventually. However, owing to their non-renewable nature, fossil fuels account for more than 80% of the power consumed at present, the combustion of which inevitably soars greenhouse gases, even dangerous gases, leading to climate change [1]. Against the backdrop of this, efforts are therefore essential to improve the energy storage abilities of “clean energy” devices, for example, supercapacitors (SCs), metal-ion batteries, metal batteries, metal-air batteries (MABs), and so forth [2, 3]. It is worth noting that, despite the distinct differences in energy storage mechanisms, their electrochemical processes involve chemical reactions or physical interactions and are mainly dominated by the materials selected, while the kinetics and transport behaviors of carriers are significantly influenced by the architecture of electrodes [4]. Based on this, innovation of high-specific capacity chemistries and optimal electrode architecture design are two promising methods to fulfill the ever-increasing energy depletion supported by these advanced electrochemical energy storage (EES) devices. In the context of practical applications, it is very desirable and practically necessary to combine material chemistry exploration and electrode structure construction to satisfy the increasingly urgent requirements both from academia and industry.

Given the recent rapid upscaling of the present progress of exciting chemistries, researchers believe that electrode configuration, especially related to nano- and micro-structured (NMS) technology, enables them to unleash their performance potential in terms of achieving their long-term energy storage goal [5–8]. This should be attributed to the fact that energy performance is directly dependent on the functional materials of electrodes, including chemistries, mass loading, and active component ratios in the device. Without functional chemistry change, the deliberate design of electrode configuration, such as a thick-film electrode, enables effective enlargement of the mass loading of active materials, resulting in higher electrochemical performance per square area or per volume. Besides, adopting porous nano- and micro-structuring of electrodes is a feasible and universal approach to ensure sufficient contact and reaction between electrolyte and active materials, which effectively maximizes the active component ratio in the device, preventing the generation of dead volume, especially for thick-film electrodes [9, 10]. Despite the remarkable progress that has been made, a major bottleneck is the limited stability of NMS electrodes. In detail, during the repeated charge–discharge processes of metal-ion batteries, the NMS electrode materials suffer from huge volume changes, resulting in fracture and delamination, even collapse and pulverization, harboring challenges for the attainment of electrochemical performance and the insufficient stability of EES devices [11, 12]. Besides, it is important to note the fact that the NMS electrodes normally should be stiff enough to sustain a necessary stack pressure (0.1–10 MPa) during device assembly (coin- and punch-cell) to ensure intimate contact between cell components as well as maintain NMS features.

**Fig. 1:**
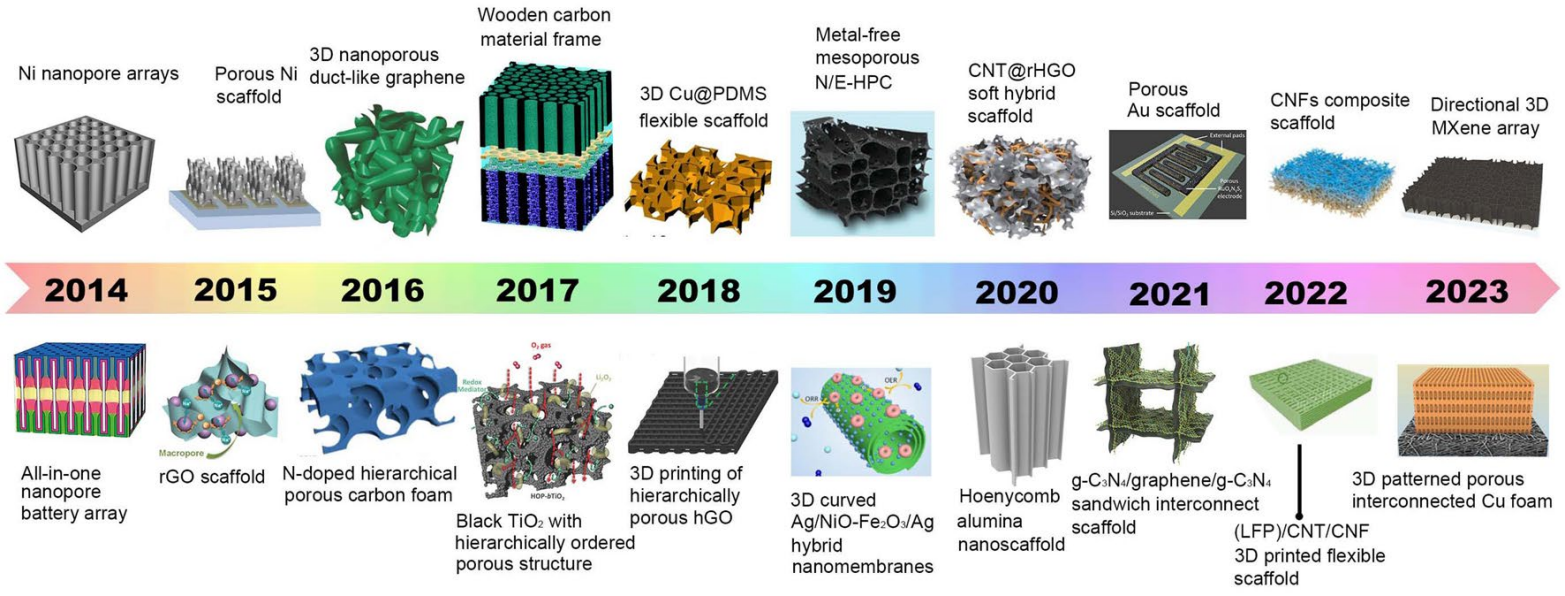
Brief development timeline featuring representative 3D NMS scaffolds for SCs, AIBs, metal batteries, and MABs spanning the past decade. Inset images: Ni nanopore arrays. Reproduced with permission from Ref. [27]. Copyright 2014, Wiley–VCH; All-in-one nanopore battery array. Reproduced with permission from Ref. [28]. Copyright 2014, Springer Nature; Porous Ni scaffold. Reproduced with permission [29]. Copyright 2015, National Academy of Sciences; rGO scaffold. Reproduced with permission from Ref. [30]. Copyright 2015, Wiley–VCH; 3D nanoporous duct-like graphene. Reproduced with permission from Ref. [31]. Copyright 2016, Wiley–VCH; N-doped hierarchically porous carbon foam. Reproduced with permission from Ref. [32]. Copyright 2016, Elsevier; Wooden carbon material frame. Reproduced with permission from Ref. [33]. Copyright 2017, Royal Society of Chemistry; Black TiO_2_ with hierarchically ordered porous structure (Reproduced with permission from Ref. [34]. Copyright 2017, Wiley–VCH; 3D Cu@PDMS flexible scaffold. Reproduced with permission from Ref. [35]. Copyright 2018, Springer Nature; 3D printing of hierarchically porous hGO. Reproduced with permission from Ref. [21]. Copyright 2018, Wiley–VCH; Metal-free mesoporous N/E-HPC. Reproduced with permission from Ref. [36]. Copyright 2019, Wiley–VCH; 3D curved Ag/NiO-Fe_2_O_3_/Ag hybrid nanomembranes. Reproduced with permission from Ref. [37]. Copyright 2019, Elsevier; CNT@rHGO soft hybrid scaffold. Reproduced with permission from Ref. [38]. Copyright 2019, Wiley–VCH; Honeycomb alumina nanoscaffold. Reproduced with permission from Ref. [18]. Copyright 2020, Wiley–VCH; Porous Au scaffold. Reproduced with permission from Ref. [39]. Copyright 2021, American Chemical Society; g-C_3_N_4_/graphene/g-C_3_N_4_ sandwich interconnect scaffold. Reproduced with permission from Ref. [40]. Copyright 2021, Wiley–VCH; CNFs composite scaffold. Reproduced with permission from Ref. [41]. Copyright 2022, Elsevier; (LFP)/CNT/CNF 3D printed flexible scaffold. Reproduced with permission from Ref. [42]. Copyright 2022, Wiley–VCH; Directional 3D MXene array. Reproduced with permission from Ref. [43]. Copyright 2023, Wiley–VCH; 3D patterned porous interconnected Cu foam. Reproduced with permission from Ref. [44]. Copyright 2023, Springer Nature

Based on these considerations, so far, quite a few NMS scaffolds have emerged toward realizing both the robustness and electrochemical performance enhancement of electrodes. Other than considering materials that inherit robustness properties, from a structural point of view, the stereoscopic geometrical design of NMS scaffolds (shape, periodicity, and porosity) has been more spotlighted, attributing to its profound effect both on physical and chemical properties for ultimate EES device performance [13–17]. Different from conventional electrodes, in which active materials, conductive additives, and binders are slurry coated onto metallic foil current collectors, three-dimensional (3D) porous interconnect NMS scaffolds make electrodes possess versatile advantages in manifold aspects. One promising advantage is that the superior surface areas endow NMS scaffold devices with a wealth of chemical reaction sites on the surface or near surface with better functionality for related processes, such as pseudocapacitance, diffusion-limited intercalation processes, oxidation–reduction reactions, and so forth. It is imperative to point out that NMS scaffold is different from NMS material, for which NMS scaffold is dedicated to transforming foil current collectors into 3D NMS mode with a specific surface area two or even three orders of magnitude higher than that of NMS material [18, 19]. Moreover, its unique advantages also bring impressively positive effects in other aspects. On the one hand, NMS scaffolding is a more straightforward and effective method to endow the macroscopic materials with microscopic properties, especially for functional NMS materials that are difficult to construct self-supporting structures. On the other hand, as a smart and versatile strategy, NMS scaffolding makes sure transforming NMS materials directly into hierarchical NMS materials with multiscale architecture porosity [13, 16, 20–22]. Meanwhile, adopting the NMS scaffold approach is a smart strategy for utilizing the third dimension to load more active materials without conductive additives and binders, resulting in maximizing the ratio of active components successfully and preventing sacrificing charge transfer kinetics simultaneously between the electrodes [16, 18]. Another point is that, benefiting from macro-pores stereoscopic architecture provided shorten diffusion pathways and some conductive network provided transport channels, NMS scaffold-based electrodes are ascertained to exhibit better kinetics and transport behaviors, endowing the advanced devices with boosting capabilities in electrochemical activity and charge–discharge kinetics [7]. Simultaneously, NMS scaffold generally possesses superior mechanical properties to maintain NMS features during the device assembly process, while considerable pore volumes enable electrodes to provide sufficient accommodation for expansion and strain relaxation with better cyclic capability during practical applications [18]. What is more, thanks to the significant progress of nano- and micro-fabrication technology (e.g., templating, 3D-printing, etc.) and assembly modes such as magnetic-assisted oriented assembly, it is possible to tailor NMS scaffolds with precise control at the multiscale, which endows NMS scaffolds with flexible design ability to control the ultimate properties of EES electrodes [23–26]. In the past decade, much progress in NMS scaffold designs and fabrication techniques has realized comprehensive performance enhancement of EES, offering new opportunities to impose the genuine potential of materials toward higher energy density. Important development of 3D NMS scaffold design for advanced EES devices is depicted in Fig. 1. Despite the fact that the selected NMS scaffolds possess different types of storage mechanisms, their similar electrochemical processes meet the same requirements from a structural point of view, which prompts our intention to summarize and highlight the structure-functions of NMS scaffolds in arenas as diverse as morphologies and electrochemical performance.

This review aims to present a comprehensive summary of NMS scaffolds in superiority, fabrication, and update developments for advanced EES devices in arenas as diverse as SCs, alkali metal-ion batteries (AIBs), the anode of metal batteries, and the cathode of MABs. The superiorities and the corresponding challenges are pointed out systematically. Eventually, the corresponding potential solutions are discussed, and the design principle of NMS scaffolds is pointed out, as well as key perspectives for the future in this realm. The remainder of this review is categorized and arranged as follows: In Sect. 2, the fundamentals of NMS scaffolds are defined, and their superiorities for EES applications are discussed in detail. In Sect. 3, NMS scaffolds are classified into flexible, hard stochastic, and hard periodic, and the corresponding preparation techniques are explained. Updated 3D NMS scaffold-based electrodes for SCs, AIBs, anode of metal batteries, and cathode of MABs are evaluated in Sect. 4, respectively. Finally, Sect. 5 summarizes the existing challenges and potential solutions.

## Fundamentals and Superiorities of NMS Scaffolds in EES

### Fundamentals of NMS Scaffolds

Despite the great achievements of NMS materials in the field of EES, further enhancements are needed to achieve the desired performance for industrial applications. Generally speaking, material and structure are the two main aspects that affect the physicochemical properties. Thanks to the efforts of researchers in recent decades, the performance of various energy storage materials has basically approached the limit of theoretical values. At the same time, there is an academic consensus that the potential of materials can be further exploited through the utilization of the 3D structure to endow them with unique chemical and physical properties, attracting more and more attention with the development of nano- and micro-science in recent years.

3D NMS scaffolds are skeleton structures with dimensions on the nanometer or micrometer scale that are capable of realizing complementary and facilitating roles for the main mechanism of application. To avoid ambiguity, the NMS scaffolds in this paper refer to 3D interconnected porous NMS scaffolds. In general, materials for 3D NMS scaffolds include, but are not limited to, carbon materials and their isomers, metallic materials, ceramic materials, semiconductor materials, polymers, and so forth [18, 39, 45, 46]. 3D NMS scaffolds involve homogeneous and heterogeneous materials, i.e., in addition to a single material, multiple materials can be combined to frame more complex heterogeneous scaffolds. Only for heterogeneous 3D NMS scaffolds it is necessary to ensure the stability and homogeneity of the phase interface in order to maintain low interfacial resistance. The selection of materials for 3D NMS scaffolds needs to comply with the following requirements: both to have certain mechanical properties to provide stable support for the active material and to remain inert in the electrolyte operating conditions. Although the 3D NMS scaffold is not a direct participant in the EES reaction, either unstable mechanical properties or corrosion by the electrolyte can produce irreversible side reactants or collapsed and crushed structures, which can greatly affect the electrochemical performance of the whole device.

**Fig. 2:**
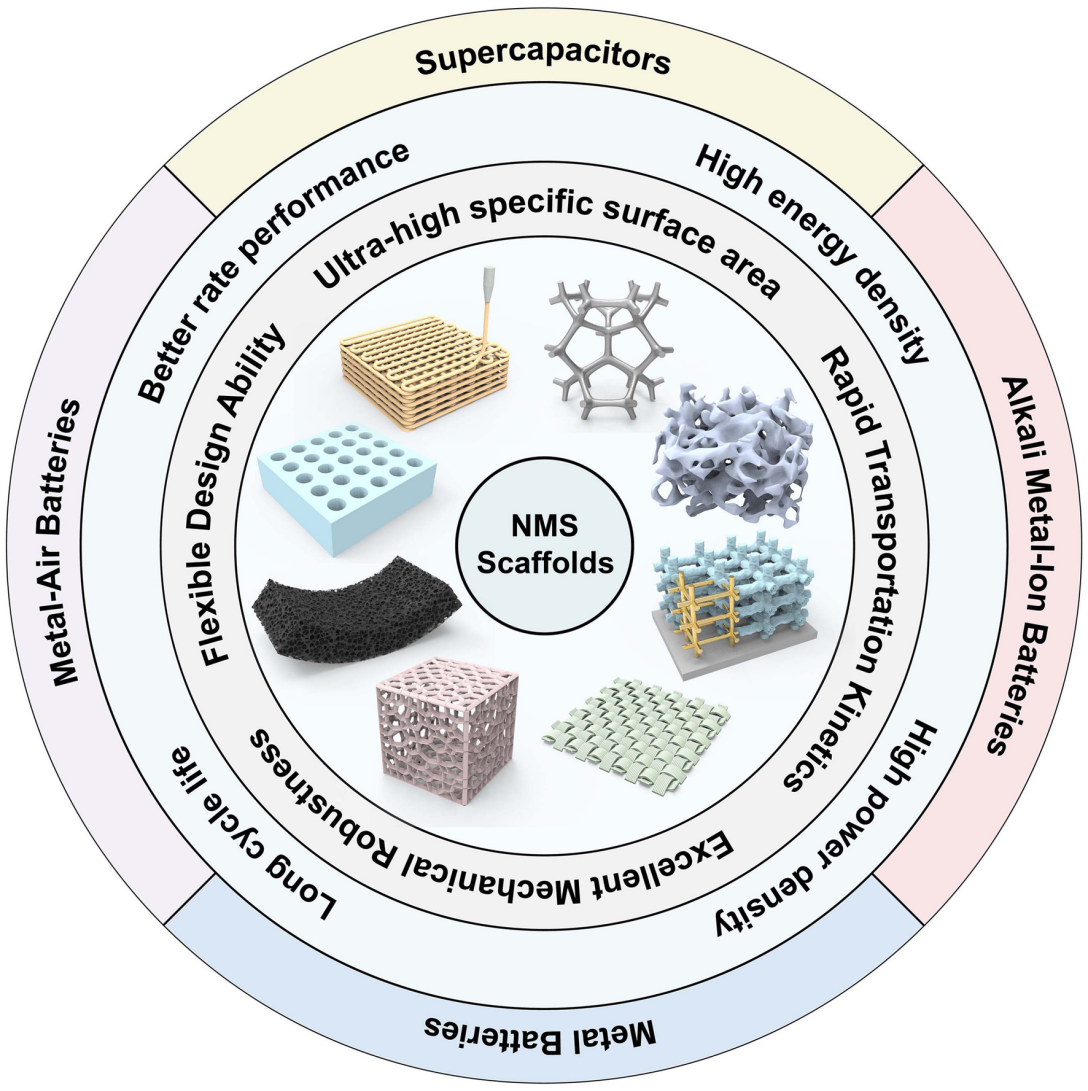
Schematic illustration of updated 3D interconnection NMS scaffolds with superiorities for high-performance EES devices over the past decades

Compared to the material issues, structural aspects of NMS scaffolds doubly have attracted tremendous attention [47]. As shown in Fig. 2, there are various updated 3D interconnection NMS scaffolds with superiorities for high-performance EES devices over the past decades. One of the impressive features is the direct design and construction of 3D NMS scaffolds, which fully demonstrates its flexible design ability. Through rational structural design, predictable 3D NMS scaffold-based electrodes can be prepared according to the requirements of different applications, which can effectively improve the performance of the devices. This has, in turn, enabled further uncovering of the electrochemical behaviors at a more fundamental level due to the structure–function relationship for electrochemical reactions when combined with simulations. This is of great significance for EES technologies, structural engineering techniques, and simulation studies in related fields, while also being able to fully stimulate the application potential of active materials to cope with the growing energy demand.

### Superiorities of NMS Scaffolds in EES

**Fig. 3:**
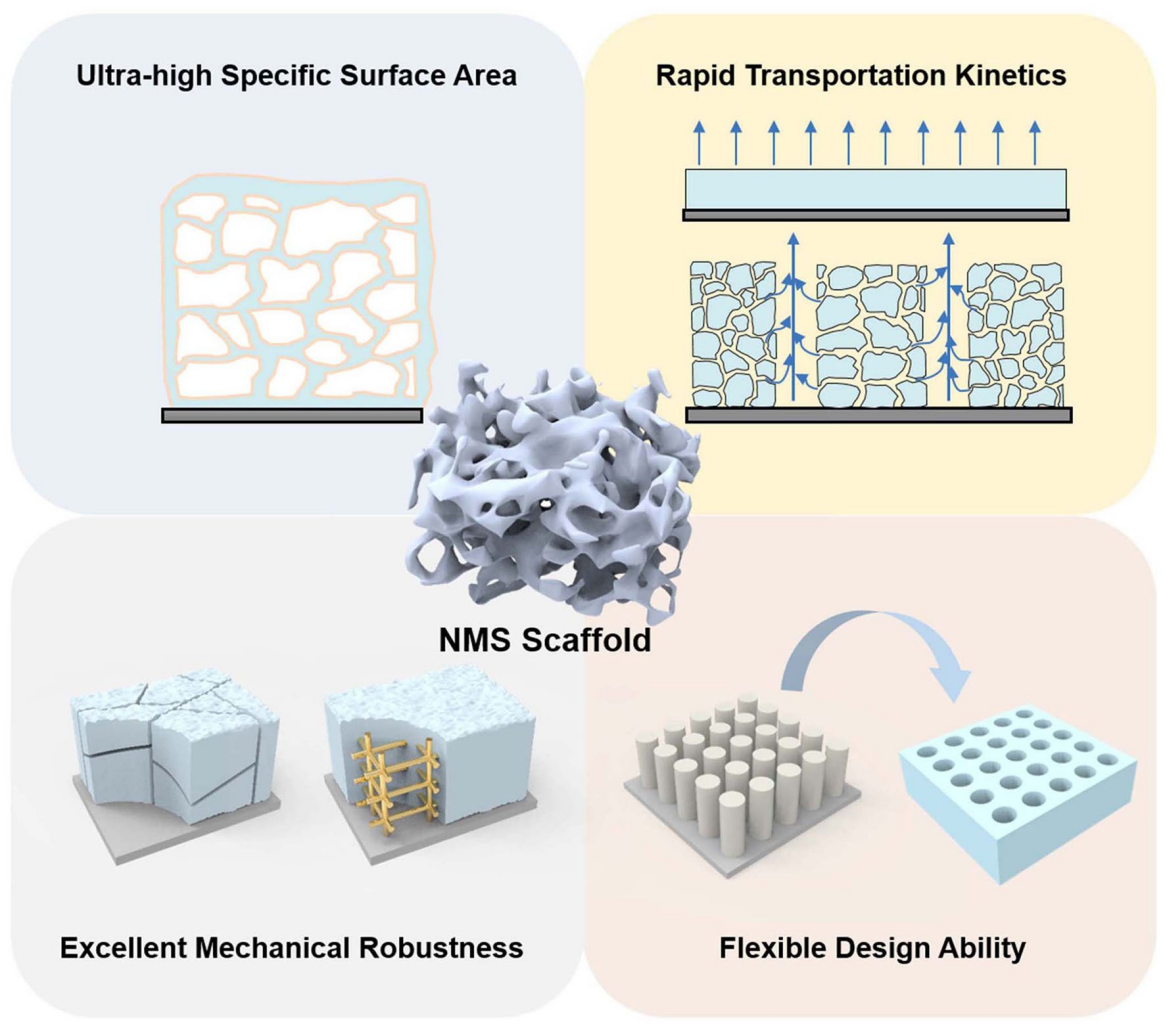
Schematic illustration of the advantages of 3D NMS scaffolds in terms of chemical reaction, ion transport, mechanical robustness, and design ability

The EES devices discussed in this paper mainly include supercapacitors, AIBs, the anode of metal batteries, and the cathode of MABs. Despite different energy storage mechanisms, the 3D NMS scaffolds share similarities in terms of chemical reactions, kinetic transport, and mechanical properties for electrochemical processes. Given the increasing energy demand, enormous efforts have been devoted to the development of high EES devices with both high-energy and power densities and a long cycling life span. Even though increasing the active material loading to accomplish the preparation of thick electrodes is an attractive strategy, a variety of unmet challenges still remain. In the case of AIBs, for example, to avoid collapse or pulverization of the active material film during the processing process as well as charge–discharge cycles, the volume ratio of conductive adhesive and binder in the device has to be increased without capacity contribution, compromising energy storage ability. Similarly, conventional thick electrodes usually succumb to sluggish charge transfer kinetics, which leads to a degradation of the rate performance and a limited energy density enhancement. In addition, cracking, pulverization, or delamination of the electrodes with the collector due to uneven internal high stress distribution during charging and discharging of thick electrodes can lead to serious degradation of the energy storage device performance and shortening of the cycle life [11, 12]. Therefore, the results obtained are contrary to the original intention. It is important to always keep in mind that high energy density, high power, and long cycle life have always been the scientific pursuits of energy storage. In contrast, electrodes based on 3D NMS scaffolds have attracted much attention due to their superior physical and chemical properties and are considered to be a strategy with the potential to comprehensively enhance the performance of EES devices. In this regard, the advantages of 3D NMS scaffolds in terms of chemical reaction, ion transport, mechanical robustness, and design ability are summarized in Fig. 3 as follows.

#### Ultra-high Specific Surface Area

The continuous porous structure of 3D NMS scaffolds allows them a large specific surface area, which is important for EES devices. Inherently, the large specific surface area can provide more electrochemical reaction interfaces and reaction active sites, which means that it can accommodate more charges and reach a higher energy density [48]. Intuitively, the large specific surface area can increase the contact area between the electrodes and the active material, thus accelerating the transfer speed between electrons or ions and the active material, which helps to improve the multiplicity performance of the energy storage device [49]. Moreover, the large specific surface area helps to spread out the distribution of electrons and ions in the electrodes, preventing the occurrence of side reactions caused by uneven local current density and improving the stability of the energy storage device.

#### Rapid Transportation Kinetics

Transport kinetics in EES refers to the transport behavior of electrons and ions. In electrodes based on 3D NMS scaffolds, the transport path of electrons is based on a 3D conductive collector network, whereas the transport path of ions relies on the negative space that can be penetrated by the electrolyte left by the electrode. In electrodes based on interconnected porous 3D NMS scaffolds, the presence of collectors is divided into two cases, including the conductive scaffolds acting directly as collectors and the scaffolds themselves being unconducive, integrating a conformal conductive thin film on their surfaces acting as collectors. Both cases are complete with a 3D interconnected porous structure, which also ensures a convenient electron transport pathway. Ion transport, on the other hand, drives the attention of researchers. Over the past decade, 3D nano- and micro-structuring have been recognized as an effective strategy for tuning ion transport mobility and selectivity. On the one hand, nanostructures can modulate the local electric field, regulate the ion concentration in the electrolyte, and alleviate the problem of slow local kinetics. On the other hand, nano- and micro-structures can shorten the ion diffusion distance. The diffusion time of ions in nanostructures can be described by Einstein's formula *T* = *λ*^*2*^*/*Di (where *λ* denotes the lateral size of the nanostructure and Di is assumed to be independent of nanosize) [49, 50]. That is, under the same conditions, the size reduction will greatly shorten the ion transport time. Because of this, in electrodes based on 3D NMS scaffolds, the thin film-shaped active material is able to complete the electrochemical reaction with the electrolyte quickly. Another point to note is the tortuosity of the 3D NMS scaffolds. According to the expression of effective ionic conductivity, *D*_eff_ = *D(ε/τ)*, where *ε* represents porosity, *τ* is tortuosity, and *D* means intrinsic ionic conductivity; it can be concluded that the effective ionic conductivity is inversely proportional to the tortuosity of the 3D scaffold [9, 51]. In other words, for 3D NMS scaffolds, utilizing scaffolds with low tortuosity in the vertical direction to ensure a low-tortuosity transport path for the electrolyte can effectively enhance the electrochemical performance of the electrode.

#### Excellent Mechanical Robustness

As previously described in Sect. 2.2, simply increasing the thickness of the active material brings about a serious loss of mechanical properties, which in turn shortens the cycling life of the device. The preparation of electrodes using 3D NMS scaffolds can effectively solve this problem. For example, 3D NMS scaffolds made of one-dimensional materials such as carbon cloth, carbon fiber, and other woven or stacked materials have excellent mechanical toughness and can meet the electrochemical and mechanical requirements of flexible wearable devices [52]. Of course, the material and structure of 3D NMS scaffolds are the decisive factors for their mechanical properties. The influence of structure on mechanical properties is more fascinating than that of material. The recently emerging honeycomb structure, with its in-plane cells in ordered or disordered two-dimensional arrays and parallel stacking in the out-of-plane direction, is characterized by a periodic topology assignment and thus has high specific stiffness and strength and specific energy absorption [18, 53]. Specifically, for in-plane stresses, the honeycomb absorbs them through bending deformation of the cell walls and plastic hinges at the cell-wall junctions; out-of-plane stresses are absorbed through cell wall buckling and membrane deformation. Cells are Voronoi honeycombs when they are disordered in both size and shape, and foam structures when they are also disordered in the out-of-plane direction. Thus, the introduction of suitable NMS scaffolds is certainly going to enhance the mechanical properties of electrodes. For the mechanical properties of NMS scaffold, both toughness and strength properties are influenced by several factors: (1) the basic mechanical properties of the scaffold material; (2) the relative density of the scaffold, which is the ratio of the density of the porous scaffold to the density of the corresponding bulk material [47]; (3) the geometry of the scaffold and holes; and (4) the dimensions of the scaffold in terms of porosity, pore size, and wall thickness. For example, thicker scaffolds are generally more likely to resist some degree of damage, but may also compromise flexibility. Moderate porosity improves the bending capacity of the scaffold, but excessive porosity will undoubtedly reduce its strength. Therefore, application requirements should be considered from various angles when designing the scaffold construction. When selecting the appropriate 3D NMS scaffolds for specific applications, it is necessary to consider not only the high stresses inside the electrodes due to the volume expansion of the active materials, side reactions, and inhomogeneous current distributions during the charging and discharging process, but also the mechanical stresses that need to be withstood during the device integration and encapsulation process [47, 54].

#### Flexible Design Ability

The structure of 3D NMS scaffolds determines the structure of electrodes to some extent, especially for electrodes with conformal thin-film active materials. Therefore, it is a very effective strategy to design the electrode structure and performance by designing and tuning the 3D NMS scaffolds. Due to the rapid development of processing technologies and integration processes in recent years, we have progressed from simply utilizing NMS scaffolds in nature to directly designing and preparing desired NMS scaffolds according to different application conditions. The main preparation processes, including the "top–down" template method and the "bottom–up" 3D printing technology, have been at the center of research and practical applications. The ability to prepare precisely defined 3D NMS scaffolds, and thus precisely defined electrodes and devices, is of great significance, both in terms of performance enhancement and the understanding of the relationship between the relevant structure and electrochemical properties in conjunction with simulation techniques [55–58]. This, in turn, will help researchers design and prepare more rational EES devices with better performance. It is important to note that high energy and power densities, good multiplicity performance, and cycle life are always the relentless pursuits of EES devices. Therefore, the design principle of 3D NMS scaffolds, which are complementary and useful to the main application mechanism, is to maintain or increase the EES capacity per unit of active material while minimizing the ratio of inactive components.

Based on the above unique advantages, NMS scaffolds are increasingly employed in the next generation of EES devices. In the next chapter, we summarize the main preparation methods and characteristics of NMS scaffolds.

## Classification and Preparation of NMS Scaffolds

**Fig. 4:**
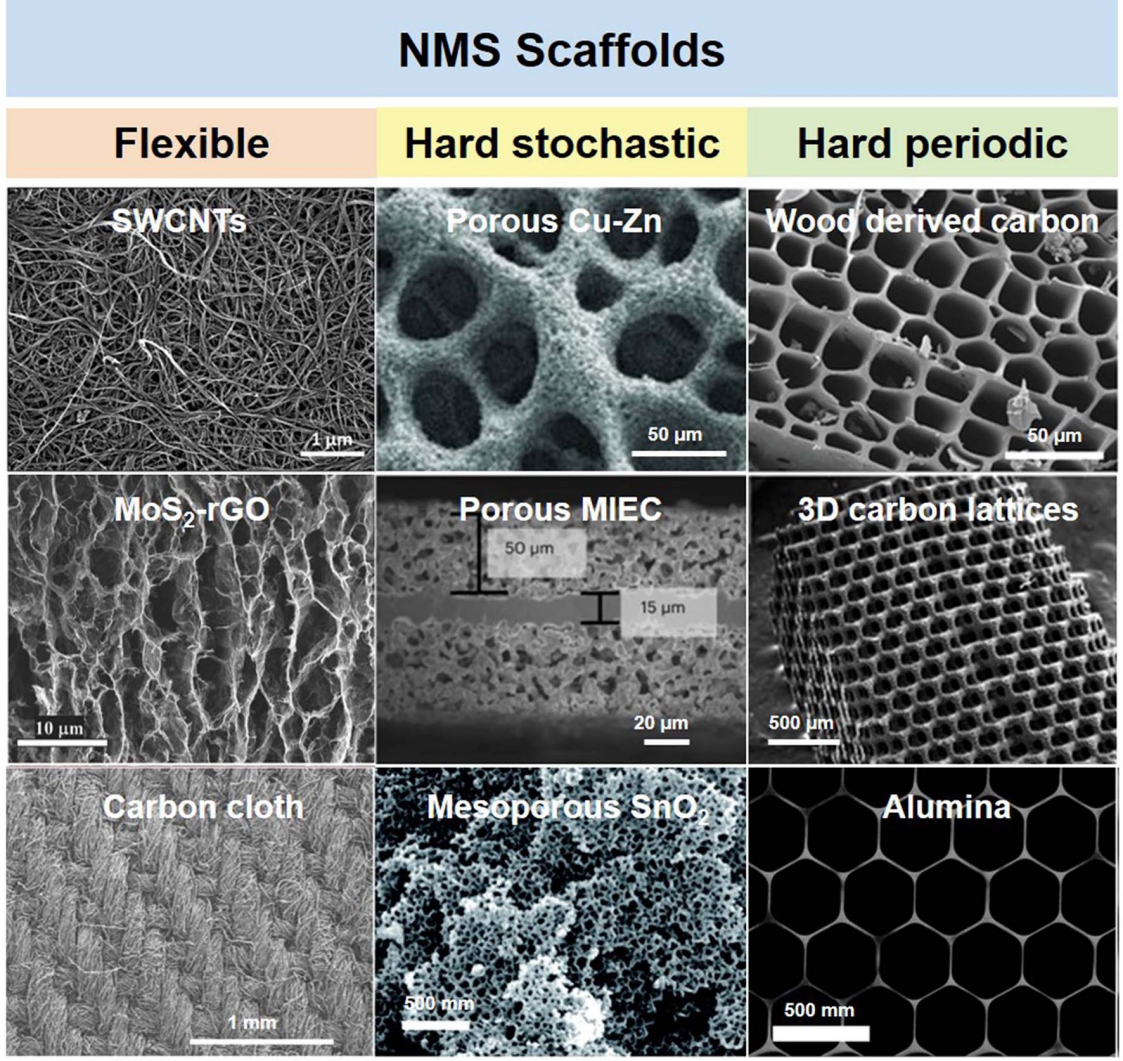
Classification and typical structure of NMS scaffolds. Inset images: single-wall carbon nanotubes (SWCNTs). Reproduced with permission from Ref. [59]. Copyright 2023, Wiley–VCH; MoS_2_-rGO. Reproduced with permission from Ref. [60]. Copyright 2017, Wiley–VCH; Carbon cloth. Reproduced with permission from Ref. [61]. Copyright 2017, Springer Nature; Porous Cu–Zn. Reproduced with permission from Ref. [62]. Copyright 2015, Wiley‐VCH; Porous mixed ion- and electron-conducting garnet (MIEC). Reproduced with permission from Ref. [63]. Copyright 2023, Springer Nature; Mesoporous SnO_2_. Reproduced with permission from Ref. [64]. Copyright 2017, Royal Society of Chemistry; Wood derived carbon. Reproduced with permission from Ref. [36]. Copyright 2019, Wiley–VCH; 3D carbon lattices. Reproduced with permission from Ref. [65]. Copyright 2022, Wiley–VCH; Alumina. Reproduced with permission from Ref. [18]. Copyright 2020, Springer Nature

As described in the previous section, porous NMS scaffolds offer several advantages to enhance the efficiency and stability of energy storage devices. For this reason, researchers have adopted many advanced preparation methods in order to design and prepare NMS scaffolds with a variety of materials and structures to meet the needs of different working conditions. In order to summarize, NMS scaffolds are classified into three main categories (shown in Fig. 4): flexible NMS scaffolds, hard stochastic NMS scaffolds, and hard periodic NMS scaffolds, and their main preparation methods and features are introduced, respectively.

### Flexible NMS Scaffolds

Portable electronic devices have disrupted the traditional lifestyle and have led to a significant improvement in the quality of life. Correspondingly, the demand for wearable energy storage devices is increasing. The application of flexible NMS scaffolds plays an important role in the development of wearable devices. Flexible NMS scaffolds can be categorized into organic and inorganic materials. Organic materials are foam structures composed of a variety of polymers (e.g., polyurethane [66], Melamine [67]). Among them, there are mainly chemical and physical methods for the preparation of polyurethane foam. However, whether it is a chemical method or a physical method, the principle is to generate gas to make the material expand and then solidify to form a foam structure. The pore size of polyurethane foam prepared by the chemical method is not uniform, and the structural stability is poor, which will lead to stress concentration, thus affecting the overall physical properties of the material. The physical preparation method has good structural stability and is more environmentally friendly, but it is difficult to realize large-scale production. Inorganic materials are mainly bendable network structures (such as carbon cloth [68], graphene network [69], etc.) constructed from low-dimensional materials through various assembly methods. In addition to the excellent flexibility of the material itself, the spacing between the materials allows for a certain degree of relative displacement, resulting in good bending ability so that the scaffold remains stable through multiple bends and deformations. Researchers have used a variety of approaches to realize the construction of flexible NMS scaffolds. Generally speaking, it is a relatively convenient strategy to construct flexible scaffolds through the self-assembly of materials. Physical or chemical interactions of independent individuals driven by magnetic, electric, inertial, and hydrodynamic forces result in spontaneous assembly into a stable state [4]. Not only is it a simple operation, but this strategy also allows for extensive hybridization of the scaffolds with other inorganic elements or compounds at the same time, thus taking full advantage of their functionality [70]. Based on this, the freeze casting method has been developed in order to realize further design of the scaffold morphology. The freeze casting method constructs a porous network system by controlling the solidification of the liquid phase solution and sublimating the solvent (usually ice); thus, it is also referred to as the ice template method. Moreover, the microstructure of the scaffolds, such as porosity and pore morphology (lamellar, honeycomb, radial, etc.), can be customized by adjusting the process parameters [23]. Since its development, freeze casting has become an important and versatile method for the preparation of porous scaffolds, mostly used for assembling graphene, carbon nanotubes, and other low-dimensional materials. Textile technology is equally capable of achieving control over the scaffold structure. Although the modulation scale is larger, the structure of the resulting fabric is more stable and durable, and it is equally widely used as a flexible porous scaffold.

### Hard Stochastic NMS Scaffolds

Hard NMS scaffolds also occupy an important position in the electrodes of energy devices. Although its flexibility is relatively poor, its considerable mechanical strength can stabilize the electrode structure to avoid volume change and cracking of the active material, thus significantly enhancing the service life of the electrode. And according to the shape and distribution law of its pores, it can be categorized into random scaffolds and ordered scaffolds. Typical hard stochastic NMS scaffolds include disordered interconnected network structures composed of metals [39, 71, 72] and metal compounds [34, 37]. Although the metal network (foam) also possesses a certain degree of flexibility and bendability, it inevitably cracks or even breaks after many bends. Therefore, we distinguish it from flexible scaffolds. Common methods for synthesizing hard stochastic NMS scaffolds include high-temperature vacuum distillation, chemical/electrochemical etching, soft templates, and sol–gel methods. High-temperature vacuum distillation utilizes the difference in boiling points of different metallic elements to remove certain elements from the alloy material to obtain a continuous porous metallic scaffold. Similarly, chemical/electrochemical etching is used to etch a metallic material into a porous structure through an acidic solution or electrochemical process. Both approaches process the bulk metal to obtain randomized porous NMS scaffolds, but suffer from the disadvantage of huge loss of raw materials. The soft template method involves immersing a soft material made of polymers or surfactants into the scaffold precursor and curing it. Then, the soft template is removed by chemical reaction or heat treatment to make the scaffolds loose and porous. This method can prepare a large area of continuous porous network structure, but the stability of the material in the process of removing the template is required to be high. The sol–gel method is a method that utilizes a sol and gel process in conjunction with heat treatment to form a porous network structure. By controlling the process of sol–gel, the final morphology of the scaffolds can be regulated. However, the preparation process may require some toxic organic solvents and may introduce inactive substances to increase the extra mass of the scaffold.

### Hard Periodic NMS Scaffolds

It is well known that the physical and chemical properties of materials are highly influenced by their microstructure [73, 74]. Therefore, attempts have been made to achieve in-depth study and utilization of material properties through precise control of the nanomaterial structure. Likewise, controllable periodic NMS scaffolds show great promise in promoting energy storage due to their unique advantages. On the one hand, regularly arranged periodic structures usually have better mechanical properties, and the scaffolds are excellent in terms of structural support and loading. On the other hand, orderly arranged regular pore structures offer significant performance advantages in charge transport, ion distribution modulation, and substance transportation and can even satisfy several needs at the same time [9]. For this reason, various strategies have been employed to fabricate periodic NMS scaffolds. Porous wood scaffolds with oriented arrangement of pores can be obtained by pretreating (e.g., alkaline solution immersion, physical gas activation, and enzymatic hydrolysis) wood materials and then carbonizing them. The advantages are that they are cost-effective and environmentally friendly, and their pore size can be regulated to a certain extent, but the homogeneity and periodicity of the pores are inferior. Highly ordered NMS scaffold structures can be obtained by means of 3D printing, photolithography, and template-mediated growth. Photolithography requires uniform spin coating of photoresist onto the material surface, followed by patterning of the material by a combination of electromagnetic radiation (e.g., UV or X-rays) and mask plates. This technique is capable of obtaining large-area and high-precision periodic porous structures, and the mask plate can be utilized to design a number of parameters such as shape, pore size, and spacing. However, the design accuracy is mainly limited by the precision of the photolithography machine, and it needs to be operated in an extremely clean environment. 3D printing has been receiving a lot of attention from researchers in the last few years. Almost any geometry and structure can be precisely customized by the rapid prototyping process. At the same time, the technology is applicable to a wide range of materials and allows precise control of complex internal structures with multiple parameters (porosity, pore size, etc.). The design obstacles are mainly due to the limitations of the ink preparation and the precision of the printer. Moreover, the main drawbacks of these two strategies are the stringent equipment requirements and high preparation costs. A low-cost and precisely controlled preparation method is template-mediated growth, in which the template structure guides the material to grow in a specific structure. Template intervention can control the material to inherit all geometrical features (shape, arrangement, pores, etc.) of the template, thus preparing a 3D structure identical or opposite to the template. Among them, AAO template has been widely used as a typical hard template. It can not only be employed as NMS scaffolds to support materials but also be used as templates to assist in the synthesis of NMS scaffolds for a variety of materials, showing a wide range of applications.

The preparation methods and characteristics of various types of NMS scaffolds are summarized above. In the next section, we will focus on the function of NMS scaffolds in a variety of EES devices.

## Updated NMS Scaffolds for Advanced EES

Based on the unique structural and functional advantages, NMS scaffolds have a broad application prospect in the field of EES systems. In this section, we review the representative research progress of NMS scaffolds in supercapacitors, AIBs, anode of metal batteries, and cathode of metal-air batteries.

### Updated NMS Scaffolds for Supercapacitors

Owing to their high-power density and ultra-long cycle life, SCs have been well established as promising power candidates to facilitate our life in high-power application fields, including memory backup, tramways, vehicles, etc. [75, 76]. However, compared to metal-ion batteries, the compromised energy density of SCs remains a major bottleneck, leading to their inability to achieve widespread practical application in the commercial market as alternative power sources. As known, the energy stored in SCs is defined as *E* = CV^2^*/*2 [77]. Based on variations in their actual charge storage mechanisms, SCs can be recognized into electrical double-layer capacitors (EDLCs) and pseudocapacitors. EDLCs utilize reversibly adsorbing charge through physical electrosorption at the electrode surface without any faradaic reactions. The capacitance of EDLC is described due to the formula *C*_EDLC_ = *ε*_*r*_*ε*_*o*_*S/D* (where *ε*_*r*_ and *ε*_*o*_ are the dielectric constants of the electrolyte and vacuum, respectively, *S* represents the surface area of the electrode, and *D* is the charge separation distance) [78]. This means that enlarging the surface area by tailoring the pore size and pore volume is conducive to optimizing the capacitance of EDLCs. Unfortunately, despite much effort, their maximum capacitance is still confined due to their fundamental charge storage mechanism. Distinguished from EDLCs, pseudocapacitance is a faradaic energy storage approach that exhibits promising potential for higher energy performance because its storage mechanism is mainly involved in electrosorption or redox reactions at or near the surface of electrodes [79]. The capacitance of pseudocapacitors is represented as *C*_pseudo_ = *A(nF/m)/V* (in which *A* is the surface of active material, n means the number of electrons, *F* illustrates the Faraday constant, and *V* is potential) [80]. *m* is the weight of active material, which is utilized to describe pseudocapacitive materials with gravimetric capacity. It is also possible to use surface area as a normalizing factor to evaluate the capacity performance of SCs, especially for micro-supercapacitors (MSCs) with a limited footprint area [81]. Thereafter, rational design of electrodes through NMS technology plays a significant role in unleashing the performance potential of active materials in terms of achieving comprehensive performance enhancement.

It deserves to be emphasized that NMS scaffolding is a more straightforward and effective method to endow the active materials with nano- and micro-properties, from a structural point of view, especially for some functional materials cannot transform into NMS materials. Hence, NMS scaffolds are of great feasibility to drive the inferior energy density performance because of that not only it enables offering ultra large specific surface area, its open volume architecture also exhibits excellent mass loading accommodation of active materials for pseusocapacitors. Thus, a plethora of advanced electrodes for SCs and MSCs based on representative NMS scaffolds are extracted and summarized in Table 1. To this end, following the concept that optimizing the energy density of SCs to battery level without sacrificing the capabilities of high-power density and long lifespan is practical, utilizing NMS scaffolds to construct 3D electrodes rather than traditional thick electrodes with better mass loading accommodation is a promising strategy.

**Table 1:** Electrochemical performance of SCs & MSCs based on NMS scaffolds

Electrode materials									
3D scaffolds	Active materials	Main techniques	Electrode capacitance (mF cm^−2^)	Peak energy (μWh cm^−2^)	Peak power (mW cm^−2^)	Electrolyte/cell voltage	Lifespan		Refs
SCs based on NMS scaffolds									
CNTs/rHGO	FeOOH//MnO_2_	Vacuum filtration	6000	1050	17,200		PVA LiCl/1.6 V	90.9% (3000)	[15]
rGO	Ni–Co–O//MnO_2_	3D print, Freeze-drying	327(Ni–Co–O)//69(MnO_2_)	90	–		PVA KOH/1.3 V	89.9% (10,000)	[95]
Si microtubes	MnO_2_	PL, Bosch dry etching, ALD, ED	1500	100	7.1		5 M LiNO_3_/1 V	82% (10,000)	[46]
Si microtubes	MnO_2_	PL, Bosch dry etching, ALD, ED	1500	60	10		5 M LiNO_3_/1 V	84% (10,000)	[46]
Porous GA/COF	–	Freeze-drying, Polycondensation-termination	289 (F g^−1^)	25.3 (Wh kg^−1^)	40,000 (W kg^−1^)		1 M Na_2_SO_4_/0.8 V	92% (5000)	[100]
Natural wood	Ti_3_C_2_–Ni	Slicing, Quickly frozen, Freeze-drying	930	23	–		PVA H_2_SO_4_/0.6 V	87% (5000)	[13]
Wood carbon	MnO_2_	Carbonization, ED	4155	1600	24 (W cm^−2^)		PVA LiCl/1.8 V	93% (10,000)	[33]
wood carbon	–	Enzymolysis, Carbonization	8410	210	500		6.0 M KOH/1.0 V	86.58% (15,000)	[83]
rGO framework	MnCO_3_	Immerse, LbL, Hydrothermal	320 (F g^−1^)	–	–		–	77% (5000)	[101]
LSG	MnO_2_	Laser scribing, ED	–	42 (Wh L^−1^)	–		1 M Na_2_SO_4_/2.0 V	96% (10,000)	[102]
GF-CNT	Fe_2_O_3_//CoMoO_4_	CVD, ALD	370.2(Fe_2_O_3_)	~ 74.7 (Wh kg^−1^)	~ 11.2 (kW kg^−1^)		2 M KOH/1.2 V	95.4% (50,000)	[86]
Graphene aerogel	PPY	3D print, Freeze-drying	2000	110	–		2 M KOH/0.8 V	75% (5000)	[96]
–	Graphene/MWCNT	3D print, Freeze-drying	–	53	1.54		6 M KOH/0.8 V	90% (10,000)	[97]
Graphite foam	NiCo_2_O_4_//NC	3D print, CVD	~ 3.2 (F cm^3^)	36.9 (Wh kg^−1^)	20 (kW kg^−1^)		PVA KOH/1.6 V	87.8% (10,000)	[103]
3D graphene networks	PANI	Solvothermal self-assembly	188 (F g^−1^)	–	–		PVA H_2_SO_4_ HQ/0.8 V	82% (100,000)	[104]
Polymer	rGO	3D print, Freeze-drying, ED	293.4	8	12.56		PVA KOH/1 V	96% (5000)	[16]
MSCs based on NMS scaffolds									
Si	GNW/RuO_x_	DRIE, MPECVD	–	15.1	2.49 (mW cm^−2^)	PVA H_3_PO_4_/1 V	90.2% (3000)		[105]
AuNPs	δ-MnO_2_	Chemical dealloyed, ED	922 (F cm^−3^)	24.3 (mWh cm^−3^)	295 (W cm^−3^)	1 M Na_2_SO_4_/0.8 V	88% (20,000)		[106]
SnO_2_NT	PPY//MnO_2_	AAO, ALD, ED	260 (F g^−1^)//910 (F g^−1^)	27.2 (Wh kg^−1^)	24.8 (kW kg^−1^)	1 M Na_2_SO_4_/1.7 V	80% (2000)		[107]
Porous Au	RuO_2_.xH_2_O	HBDT, ED	3250	126	7.9 (mW cm^−2^)	PVA H_3_PO_4_–H_4_SiW_12_O_40_/0.9 V	95% (2000)		[62]
Porous Au	RuO_x_N_y_S_z_	HBDT, ED	14,300	432 (mJ cm^−2^)	421 (mW cm^−2^)	PVA H_3_PO_4_–H_4_SiW_12_O_40_/0.9 V	100% (5000)		[39]
PEDOT:PSS	Mxene	3D print, LbL	–	80	3.8	PVA-H_2_SO_4_/0.8 V	83% (6000)		[108]
Ni nanopore arrays	MnO_2_	AAO, ED	570 (F g^−1^)	–	–	1 M Na_2_SO_4_/0.8 V	83% (3000)		[27]
FTO/AAO	MnO_2_	AAO, USP, ED	87.4	1.56 (mWh cm^−3^)	–	1 M Na_2_SO_4_/0.8 V	81.6% (3000)		[98]
FTO/AAO	MnO_2_	AAO, USP, ED	540	2.36 (mWh cm^−3^)	4.45 (Wh cm^−3^)	1 M Na_2_SO_4_/0.8 V	85.5% (5000)		[99]
SnO_2_/AAO	PPY//MnO_2_	AAO, ALD, ED	–	160	40 (mW cm^−2^)	EMIM-TFSI/3 V	82.5% (10,000)		[18]
Exfoliated graphite	PPY/MoO_x_//Na_0.5_MnO_2_	Electrochemical exfoliation, ED, LbL	398 (F g^−1^)	72.7 (Wh kg^−1^)	343 (W kg^−1^)	5 M LiCl/2.2 V	92.3% (10,000)		[109]
CuSe	Ni(OH)_2_	ED		5.4 (mWh cm^−3^)	833.2 (mW cm^−3^)	PVA LiCl/1 V	100% (10,000)		[110]
CNW	Hydrous-RuO_2_	PECVD, ED	1094	49	31.3	PVA H_3_PO_4_–H_4_SiW_12_O_40_/0.9 V	~ 90% (2000)		[111]
GNW	Ni(OH)_2_	MPECVD, EB, Electrochemical oxidation	33.6	2.1 (mWh cm^−3^)	5.91 (W cm^−3^)	–	96% (2000)		[112]
Porous carbon	–	MIL, ALD		3.61	1.3	PVA/H_3_PO_4_ /1 V	97% (30,000)		[113]
GF	PPY	Laser induction	2412.2	134.4	6.5	PVA H_2_SO_4_/1.3 V	95.6% (10,000)		[114]
Au pillars	GMP	Inkjet print, LbL	10	~ 1	–	PVA H_2_SO_4_/0.8 V	80% (2000)		[115]
Graphene aerogel	MnO_2_	3D print, ED	44,130	1560	–	3 M LiCl/2.2 V	92.9% (20,000)		[116]
Mxene-AgNW-MnONW-C_60_	–	3D print, LbL, Unidirectional freezing, Freeze-drying	–	19.2	58.3 (mW cm^−2^)	PVA KOH	–		[14]

PEDOT:PSS, poly(3,4-ethylenedioxythiophene):polystyrene sulfonate; DRIE, deep reactive ion etching; PL, photolithography; MIL, multibeam interference lithography; ALD, atomic layer deposition; ED, electrodeposition; GNW, graphene nanowalls; HBDT, dynamic hydrogen bubble template; USP, ultrasonic spray pyrolysis; FTO, fluorine-doped tin oxide; EMIM-TFSI, 1-ethyl-3-methylimidazolium bis(trifluoromethylsulfonyl)imide; PANI, polymer polyaniline; GMP, graphene/MnO_2_/PANI; CNW, carbon nanowalls; EB, electron beam; LSG, Laser-scribed graphene; PPY, polypyrrole; GF, graphene framework; SLM, selective laser melting; CVD, chemical vapor deposition; NC, N-doped carbon nanosheet arrays

#### NMS Scaffold Designs for SCs

**Fig. 5:**
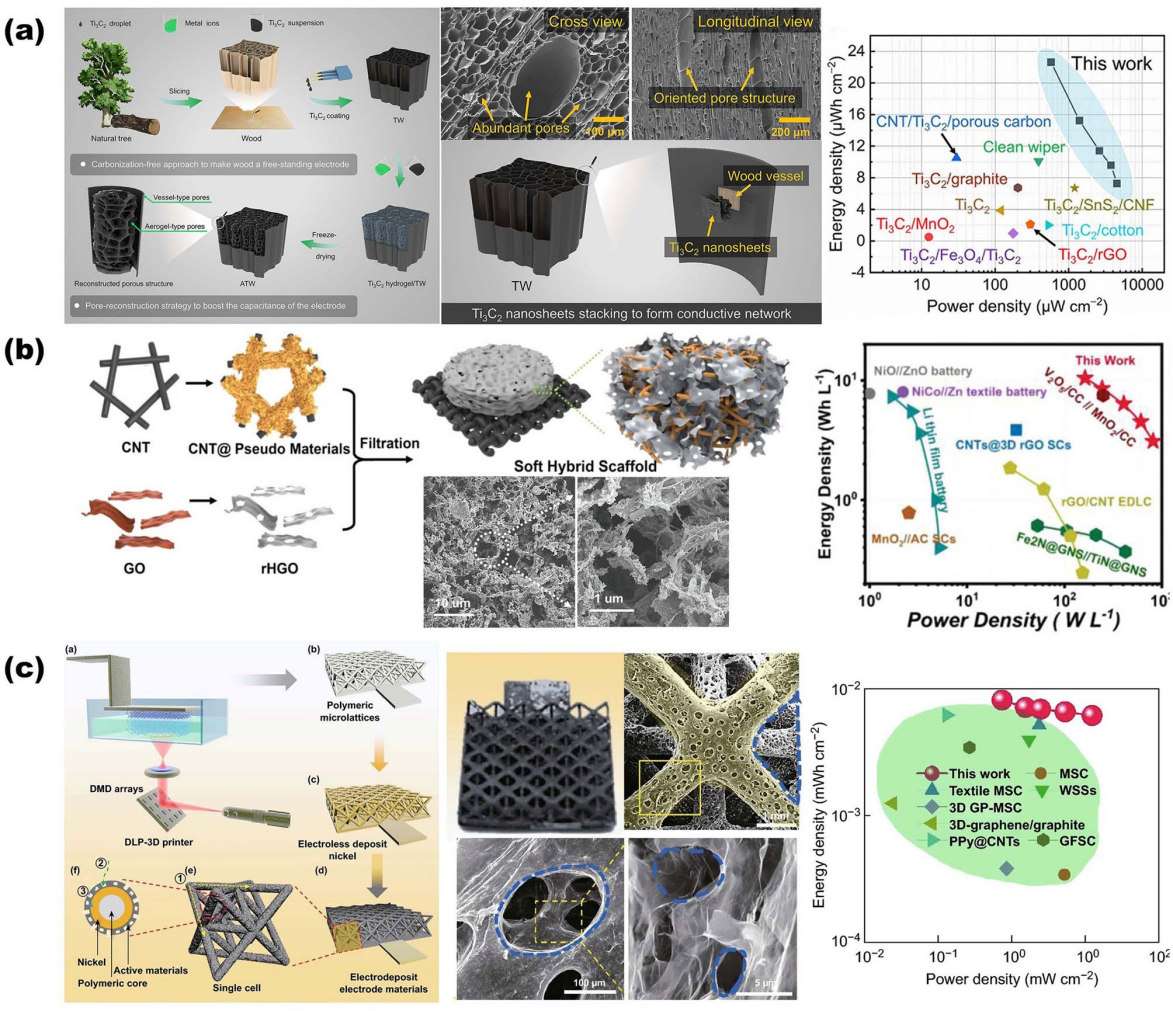
Overview of representative electrodes for SCs based on NMS scaffolds. **a** The ultimate 3D continuous network structure is based on porous wood NMS scaffold. Reproduced with permission from Ref. [13]. Copyright 2023, Wiley‐VCH. **b** Soft hybrid scaffolds consist of CNTs and rHGOs for wearable SCs. Reproduced with permission from Ref. [38]. Copyright 2020, Wiley‐VCH. **c** An octet-truss lattice polymer scaffold was synthesized via acrylate-based UV photosensitive resin. Reproduced with permission from Ref. [16]. Copyright 2019, Springer Nature

**Fig. 6:**
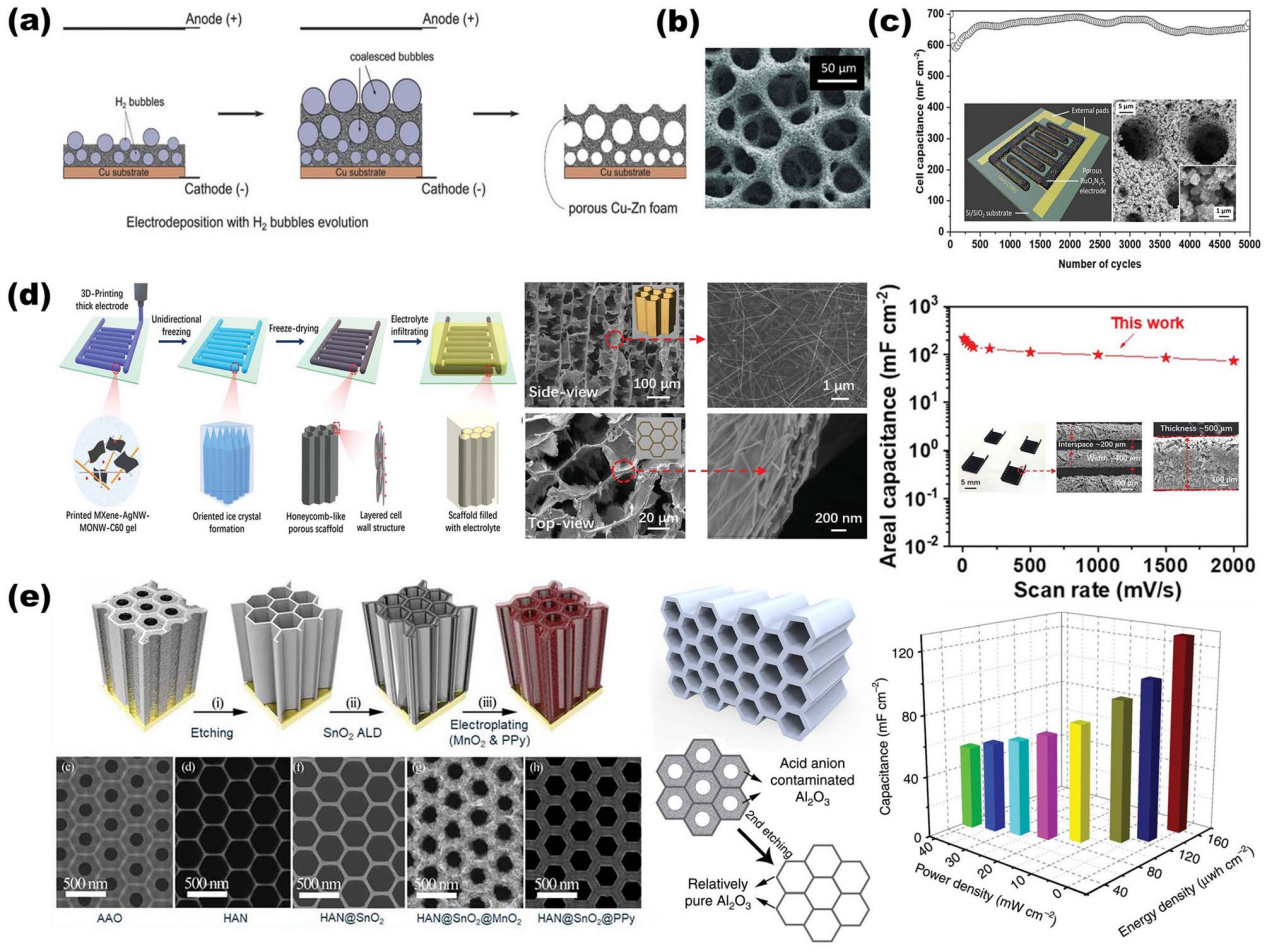
Overview of representative electrodes for MSCs based on NMS scaffolds. **a** Schematic illustration of porous Cu–Zn NMS scaffold fabricated via DHBT method. Reproduced with permission from Ref. [94]. Copyright 2018, Wiley‐VCH. **b** SEM images of the porous Au NMS scaffold. Reproduced with permission from Ref. [62]. Copyright 2015, Wiley‐VCH. **c** Electrode based on porous Au NMS scaffold with RuO_x_N_y_S_z_ as active material for MSCs. Reproduced with permission from Ref. [39]. Copyright 2021, American Chemical Society. **d** Honeycomb-like porous scaffold with fine gel (MXene‐AgNW‐MnONW‐C_60_) endowed by 3D print and freeze-drying techniques. Reproduced with permission from Ref. [14]. Copyright 2020, Wiley‐VCH. **e** Honeycomb alumina as stiff NMS scaffold with only 16 ± 2 nm cell‐wall for MSCs. Reproduced with permission from Ref. [18]. Copyright 2020, Springer Nature

During the past decades, the emerging of nano- and micro-fabrication technics combined with novel assembly modes makes it possible to tailor scaffolds in a wide range from the nanoscale to microscale, which brings up more opportunities for NMS scaffolds. Simultaneously, in contrast, the demand for advanced NMS scaffolds with unrivalled structures at nano- and micro-scale is continually facilitating the rapid development of nano- and micro-fabrication technics [82]. It is imperative to emphasize the fact that despite that NMS scaffolds act as complementary and facilitating roles during the charge–discharge energy storage process, the versatile scaffolds endowed by advanced fabrication technics significantly affect the chemical and physical properties both of the EDLCs and pseusocapacitors [47, 53, 54]. It is imperative to keep in mind that optimal NMS scaffold design is a key aspect of many of these electrodes, as it endows them with some good properties such as filtration, robustness, and kinetics. Inspired by evolved wood structure with a wealth of NMS pores at multiscale in nature, Chen et al. reported a typical all-wood-structured SCs, in which the obtained activated wood carbon directly acts as anode while MnO_2_ was electrochemical deposited onto both of the surface and its inside channels of the carbonized carbon with original thin wood membrane as separator without further treatment [33]. In this manner, natural wood directly transforms into wood carbon NMS scaffolds designed by nature via carbonization technology. Moreover, after the synergie treatment between delignification steps via chemical (e.g., a mixed solution of H_3_PO_4_, adenine, and H_2_O_2_) or biological (e.g., enzymolysis) and carbonization processes, hierarchically porous carbon-based NMS scaffolds, which feature vertical open microchannels and hierarchical nanopore properties, are successfully obtained simultaneously [83–85]. Recently, different from most parts, Chen’s group outlines an interesting strategy that straightforwardly uses natural wood as an NMS scaffold without any treatment, resulting in a hierarchically reconstructed multiscale porous structure consisting of Ti_3_C_2_ and wood vessels (as shown in Fig. 5a) [13]. The ultimate 3D continuous network structure endowed by porous wood NMS scaffold and Ti_3_C_2_ aerogels exhibits advantages such as abundant active sites with boosted capacitance and hierarchically arranged channels with accelerated electronic transportation. With these salience features, the electrode exhibits an aerial capacitance of 930 mF cm^−2^ at 0.5 mA cm^−2^ while the corresponding peak energy density of symmetric supercapacitors is 23 μWh cm^−2^. Although great progress has been made regarding wood-based NMS scaffolds for SCs, many limitations still exist, especially for flexible applications. To this end, constructing soft scaffolds based on carbon network with interconnected and porous structure is an appealing trade-off strategy to realize high energy density via mass loading enhancement of pseudomaterials without the expense of wearable attributes (for example, lightweight, mechanical flexibility and flame-retardant safety) for practical wearable SCs applications, including portable communications, wearable backups, and implantable medical devices [38, 86–89]. Case in point, Shang et al. devised a soft hybrid scaffold strategy in terms of directly vacuum filtration of carbon nanotubes (CNTs) and reduced holey graphene oxides (rHGOs), in which the properties of CNTs endow the electrode with excellent electric conductivity and mechanical robustness (Fig. 5b). Benefiting for the hierarchically porous structure, the hybrid scaffold comprising of nanotube and nanosheet, not only offers rapid ion and electron transport pathway but also exhibits good accommodate ability to load more pseudomaterials for better electrochemical performance [38]. Given the outstanding softness of all materials, the 3D network-based device holds high flexibility, enlisting fully fulfil the practical application demands for wearable SCs. Eventually, the electrode based on soft hybrid scaffold has areal capacitance of 6000 mF cm^−2^. The areal energy density and power density of the asymmetric supercapacitors are performed as 1050 μWh cm^−2^ and 17,200 mW cm^−2^, respectively, along with 90.9% capacitance retention after 3000 cycles at 50 mA cm^−2^. Other than retaining and utilizing the intrinsic nano- and micro-structure of NMS materials, as mentioned above, there is now a growing interest in emphasizing the control to precisely tailor and fabricate the NMS scaffolds in order to further uncover the structure–function relationship and make full use of active materials for performance optimization of advanced EES devices [4, 90]. In the realm of this, the 3D printing technique provides reliable bottom-up manufacturing processing that is capable of devising predictable 3D geometrically architected NMS scaffolds. Over the past decade, the current ink formulation and 3D printing technics enable the design and construction of scaffolds with nano- or micro-structures, almost any desired stereoscopic geometry at the multiscale, by being synergized with other processing technologies [91]. Specifically, Lin et al. reported a gradient porous design of graphene aerogel-graded NMS scaffold, the center-to-center ligament spacing of which is decreasing from the outer to the inner layers, facilitating sufficient diffusion of ion/electrolyte for the electrode [92]. Along with the same design concept that NMS scaffolds possess and are capable of realizing complementary and facilitating roles for the main mechanism of application, like Chen (Fig. 3a) and Lei (Fig. 4e), Xue et al. directly utilize polymer lattices as NMS scaffolds for electrodes rather than select conductive materials that act both as scaffolds and current collectors [16, 18]. As indicated in Fig. 5c, based on digital light processing (DLP) and digital mirror device (DMD) techniques, an octet-truss lattice polymer scaffold was synthesized via acrylate-based UV photosensitive resin. Furthermore, by virtue of the dynamic hydrogen bubble template (DHBT) (discussed in Fig. 6a) method, the resulted electrodes exhibit impressing structural features provided by hollow and hierarchically porous 3D NMS scaffolds, such as the fine intrinsic conductivity endowed by the rGO and NiP metal layers, fast transport for charge kinetics, ultra-large active sites, unique electrical conductivity, facilitated electron transport and ionic diffusion channels as pathways, and sufficient voids both for accommodating enough pseudomaterials and electrolyte penetration, and so forth. Ultimately, the areal capacitance performances of the rGO electrode is 293.4 mF cm^−2^ at 0.5 mA cm^−2^, together with a peak energy density of 8 μWh cm^−2^ and a power density of 12.56 mW cm^−2^ with a long lifespan (96% after 5000 cycles). As what we can concluded from the aforementioned, even as the same material (carbon or carbon derivatives), processing techniques play powerful roles in enhancing the properties of a material by virtue of optimizing their shape and size from a structural point of view.

#### NMS Scaffold Designs for MSCs

To satisfy the increasing energy storage capability requirements from implementation of the Internet of Things (IoT), despite a surge of new technologies, unmet challenges for energy density still remain for MSCs, for which they only cover a limited footprint area close to several square millimeters. Different from 2D thick electrodes with NMS materials, utilizing the capability of designable as advantage of 3D NMS scaffolds is crucial to foster strengths and circumvent the weakness of MSCs, enlisting better utilization of the space from a fundamental goal point of view [81, 93]. As an additional part within limited space, the exist of scaffold comes at the expense of some volume ratio of the device to some extent while the designable of scaffold is another key advantageous point to significantly affect the properties both of SCs and MSCs. Following this concept, it is imperative to tailor structures of NMS scaffolds precisely, even featuring the synergy among new technologies, which unleashes the potential of scaffolds in terms of boosting the energy storage performance of MSCs. When compared with more mature and precise silicon-based architectural techniques such as photolithography, the template-assisted method is another attractive, full-fledged approach in terms of tailoring the architecture and functions of NMS scaffolds. Typically, DHBT is a promising method for preparing porous single metals (e.g., Au, Ag, Pt, Cu, Sn, etc.) and binary alloy (e.g., CuAu, CuAg, PtAu, CuZn, etc.) metallic NMS scaffolds due to the advantageous both in terms of its cleanliness and ease of preparation (as shown in Fig. 6a–c) [39, 62, 94]. In this case, with numerous diverse nano- and micro-pores, such metallic NMS scaffold reported by Pech’s group suited for fully electrolyte penetration, higher active surface area value (150 times of flat Au film), ion transport facilitation, and sufficient mechanical robustness to overcome the huge volume changes during cycles [39]. Based on these advantages, accompanying with RuO_*x*_N_*y*_S_*z*_ as pseudomaterials, the high areal electrode capacitance of 14,300 mF cm^−2^ is obtained. Simultaneously, the areal energy density as well as power density of 432 mJ cm^−2^ and 421 mW cm^−2^, are yielded, respectively, coupling with the excellent cycling life (100% of initial capacitance even after 5000 cycles). In many cases, fabrication of porous scaffolds followed by in situ deposition of pseudomaterials in a conformal manner is an effective strategy. However, for some MSCs obtained by freeze casting (ice-templating), simultaneous preparation of the scaffolds and pseudomaterials in a hierarchical porous structure is possible. This ice-template assisted processing technic endowing low dimensional materials (such as nanowires, nanoplates, CNT, and etc.) with 3D structural properties and broadening their applicability. Particularly, when freeze casting is integrated with other processing routes (e.g., additive manufacturing, electrospinning, and laser etching), it is possible to obtain diverse scaffolds with nano- and micro-scale geometries. More recently, a pseudoplastic nanocomposite fine gel for 3D printed interdigitated MSCs was reported by Liang’s group. After printing step, a honeycomb-like porous scaffold was obtained by virtue of the following unidirectional freezing and freeze-drying techniques (Fig. 6d). Freeze-drying is a particularly versatile technique that enables the use of diverse assembly units for NMS scaffolds with a wide range of porosity and pore morphology (lamellar, honeycomb, etc.) tailored by chemical or physical methods [13, 14, 95–97]. Eventually, the MSCs based on honeycomb-like scaffold delivers areal capacitance of 216.2 mF cm^−2^ (at 10 mV s^−1^) and the areal energy density and power density are performed as 19.2 μWh cm^−2^ and 58.3 mW cm^−2^, respectively [14]. Given the distinct physical/mechanical attributes of honeycomb structure, anodic aluminum oxide (AAO) template method is another promising candidate strategy toward controlling the design of MSC electrodes with clear and periodic finer structure, as depicted in Fig. 6e [18, 98, 99]. Impressively, as reported by Lei’s group, this honeycomb alumina nano-scaffold (HAN) possesses surprisingly high mechanical stability despite the thickness of the cell wall is only 16 ± 2 nm, which features the synergy between the alumina physical property and honeycomb structural mechanism [18]. Meanwhile, the electrodes based on aligned HAN (conformal SnO_2_ as current collector) enabling better effective ionic transport pathways relative to random porous structures mentioned before. Finally, this MSC based on robustly stable HAN yields a high capacitance of 128 mF cm^−2^ at 0.5 mA cm^−2^, coupling with the peak areal energy densities and power density of 160 μW cm^−2^ and 40 mW cm^−2^, respectively.

Although the 3D NMS holder is not directly involved in the reaction, application-driven selection of the right material can greatly affect the overall device performance. Generally, the materials of 3D NMS scaffolds can be carbon, metal, semiconductor, ceramic, polymer, and so forth. The 3D NMS scaffolds can be used in SCs either directly as a collector or simply as a mechanical support for a thin film-shaped current collector [14, 18]. The use of metallic 3D collectors should pay attention to the voltage window range that the metal is suitable for and the stable electrolyte environment. For example, metallic collectors are typically inert in alkaline electrolyte environments, which limits the use of active materials suitable for acidic electrolyte environments. However, this problem can be perfectly solved by directly depositing active materials onto 3D MNS scaffolds that are inert or even non-conducting but have a layer of thin film-shaped collectors adapted to the environment. This provides new opportunities to enrich the choice of materials for SC collectors. At the same time, since the 3D scaffold is an additional extra part, the design of its structure should follow the lowest occupy ratio in the active volume, which is especially an important point in MSCs.

### Updated NMS Scaffolds for AIBs

Given the tremendous progress being made in developing new battery chemistries with higher specific capacity, tuning battery configurations by virtue of structural engineering is another promising candidate to satisfy the increasing demands for higher energy density in AIBs [48, 117]. Considering the significance impact of the architecture, surface area, and pore size for materials, transforming materials into nano- and micro-scale has been the central aspects of investigations and practical applications. However, there are still some materials not suitable for the transforming process by the present structuring techniques, restricting their further performance. In this perspective, utilizing 3D NMS scaffolds and introducing battery materials into a 3D volume by in situ synthesis or infiltration are a more promising strategy for unleashing the potential of battery materials [118]. In detail, 3D NMS scaffolds not only enable the endowment of materials that cannot transform into NMS materials with nano- and micro-structural properties but also offer hierarchical structure to NMS materials. It is imperative to know that beyond the advantages of inherited NMS materials, NMS scaffold generally possesses superior mechanical robustness to overcome the huge volume changes during the charge–discharge cycles, circumventing the present of pulverization and “dead” region, and further alleviating the sharp capacity decay [47, 93, 119]. Considering mass loading, NMS scaffolds can act as the host framework to accommodate the active materials in the pore space or support them in a conformal way on the surface, both of which follow the concept of enhancing the energy density at high mass loading. Moreover, whatever NMS scaffold or a conformal conductive layer on the surface of NMS scaffold is used as current collector, NMS scaffold offers a confinement effect for shortening and a homogenous charge transfer pathway, enlisting the good rate performance of batteries [18]. Considering these perspectives, utilizing NMS scaffolds to construct 3D electrodes for batteries is a promising strategy for optimizing the power density and cycle life spin without sacrificing the energy density. To this end, various advanced electrodes for AIBs and alkali metal-ion micro-batteries (AIMBs) based on representative NMS scaffolds are summarized in Table 2.

**Table 2:** Electrochemical performance of AIBs & AIMBs based on NMS scaffolds

Electrode materials									
3D scaffolds	Active materials		Main techniques	Capacity (mAh cm^−2^)	Peak energy (mWh cm^−2^)	Peak power (mW cm^−2^)	Electrolyte	Lifespan	Refs
Bs based on NMS scaffolds									
Porous carbon	ZnFe_2_O_4_		Carbonization	711 (mAh g^−1^)	–	–	LiPF_6_/EC/DEC	≈100% (400)	[122]
Porous Cu–Zn	–		HBDT, ED	≈300 (mAh g^−1^)	–	–	LiPF_6_/EC/DEC	83.3% (500)	[94]
Carbon fibers	LiNi_0.5_Mn_0.3_Co_0.2_O_2_		CVD	1500	≈380 (Wh kg^−1^)	–	LiPF_6_/EC/EMC	90.4% (500)	[87]
CNTs/CNFs	LFP//LTO		3D print, Freeze-drying,	8.4	15.2	75.9	LiPF_6_/EC/DEC	84% (10,000)	[42]
Porous carbon	LFP//Ge-N		PS template	942 (mAh g^−1^)	–	–	LiPF_6_/EC/DEC/VC	90% (200)	[123]
CB/CNF network	LFP		Self-assembly, Freeze-drying	8.8	538 (Wh L^−1^)	–	LiPF_6_/EC/DEC	91% (150)	[120]
NPC	–		Self-template	–	–	–	KPF_6_/EC/DEC	–	[15]
3D graphene	Fe_2_O_3_		Self-assembly, Freeze-drying, Ostwald ripening	–	–	–	LiPF_6_/EC/DMC	–	[124]
3D Ti_3_C_2_T_x_	LFP//Ga-In		Self-assembly	132 (mAh g^−1^)	–	–	LiPF_6_/EC/DEC/FEC	90.2% (150)	[125]
3D porous Bi	Na_3_V_2_(PO_4_)_3_//3D porous Bi		Liquid phase reduction	306 (mAh g^−1^)	116 (Wh kg^−1^)	–	Na_2_O	–	[126]
3D MXene	V_2_O_5_		Ice template	–	–	–	LiPF_6_/EC/DEC	–	[127]
3D honeycomb-like carbon grafted	PB// 3D honeycomb-like carbon grafted		Freeze-drying, Carbonization	–	113 (Wh kg^−1^)	–	KFSI/DME	–	[128]
Na_3_V_2_(PO_4_)_3_	Na_3_V_2_(PO_4_)_3_//Hard carbon		Phase separation	90 (mAh g^−1^)	–	–	NaClO_4_/EC/DEC/FEC	99% (100)	[129]
Vertically aligned graphene	LiNi_0.6_Co_0.2_Mn_0.2_O_2_//Red-phosphorus		Silica template, Coassembly	5.6	405 (Wh kg^−1^)	1224 (W kg^−1^)	LiPF_6_/EC/DMC/FEC	76.6% (300)	[130]
AIMBs based on NMS scaffolds									
Nanoporous Au	K_x_MnO_2_·nH_2_O//K_x_V_2_O_5_·nH_2_O		Chemically dealloying, ED	~ 64 (mAh cm^−3^)	~ 103 (mWh cm^−3^)	~ 600 (W cm^−3^)	0.5 M K_2_SO_4_	80% (10,000)	[121]
Porous Ni	LiMnO_2_//Sn		3D HL, PL	–	6.5 (μWh cm^−2^ μm^−1^)	3600 (μW cm^−2^ μm^−1^)	LiClO_4_/EC/DEC	80% (100)	[29]
Porous Ni	V_2_O_5_//Li		ILP, Self-assembly, ED	–	1.242 (J cm^−2^)	75.5	PEO/LiTFSI/DOL/DME	75% (200)	
Porous Ni	PIN		PL, ED, Hydrothermal	–	–	–	LiPF_6_/EC/DEC	–	[131]
Porous Ni	LiMnO_2_//Sn		3D HL, PL	–	15 (mWh cm^−2^ μm^−1^)	7.4 (mW cm^−3^ μm^−1^)	LiClO_4_/EC/DEC	64% (15)	[132]
Porous Ni	Ni(OH)_2_//Zn		Colloidal templating, ED, facile anodizing	150.1 (μAh cm^−2^)	0.26	33.8	PVA-KOH/ZnO	74.6% (1800)	[133]
3D Ni_5_P_4_ nanofoams	Li		Virus-template	677 (mAh cm^−3^)	–	–	LiPF_6_/EC/DEC	80% (100)	[134]
Porous Ni	MnO_2_		PL, PS template, ED	–	45.5 (mWh cm^−2^ μm^−1^)	5300 (μW cm^−2^ μm^−1^)	LiClO_4_/EC/DMC	–	[135]
AAO	V_2_O_5_		AAO, ALD	–	0.6 (μWh cm^−2^ μm^−1^)	0.49 (μW cm^−2^ μm^−1^)	LiPF_6_/EC/DEC	81% (1000)	[28]
Porous Ni	NH_4_CuHCF-PEDOT//Zn		ED, Coating	–	0.31	80.83	(HN_4_)_2_SO_4_/Zn(CF_3_SO_3_)_2_	–	[136]
Porous Ni	NH_4_CuHCF-PEDOT//Zn		ED, Coating	–	0.33	83.12	(HN_4_)_2_SO_4_/CuSO_4_/guar gum	94% (1000)	[136]

DEC, diethyl carbonate; EC, ethylene carbonate; EMC, ethyl methyl carbonate; PEO, poly(ethylene oxide); VC, vinylene carbonate; DMC, dimethyl carbonate; FEC, ethylene fluorocarbonate; HBDT, dynamic hydrogen bubble template; ED, electrodeposition; LFP, lithium iron phosphate; LTO, lithium titanate; PS, polystyrene colloidal nanospheres; CB, carbon black; NPC, nitrogen-doped porous carbon; PL, photolithography; HL, holographic lithography; ILP, imprint lithography; RIE, reactive ion etching; PIN, Polyimide-based nanoparticles; EMIM-TFSI, 1-ethyl-3-methylimidazolium bis(trifluoromethylsulfonyl)imide; PB, Prussian blue

#### NMS Scaffold Designs for AIBs

**Fig. 7:**
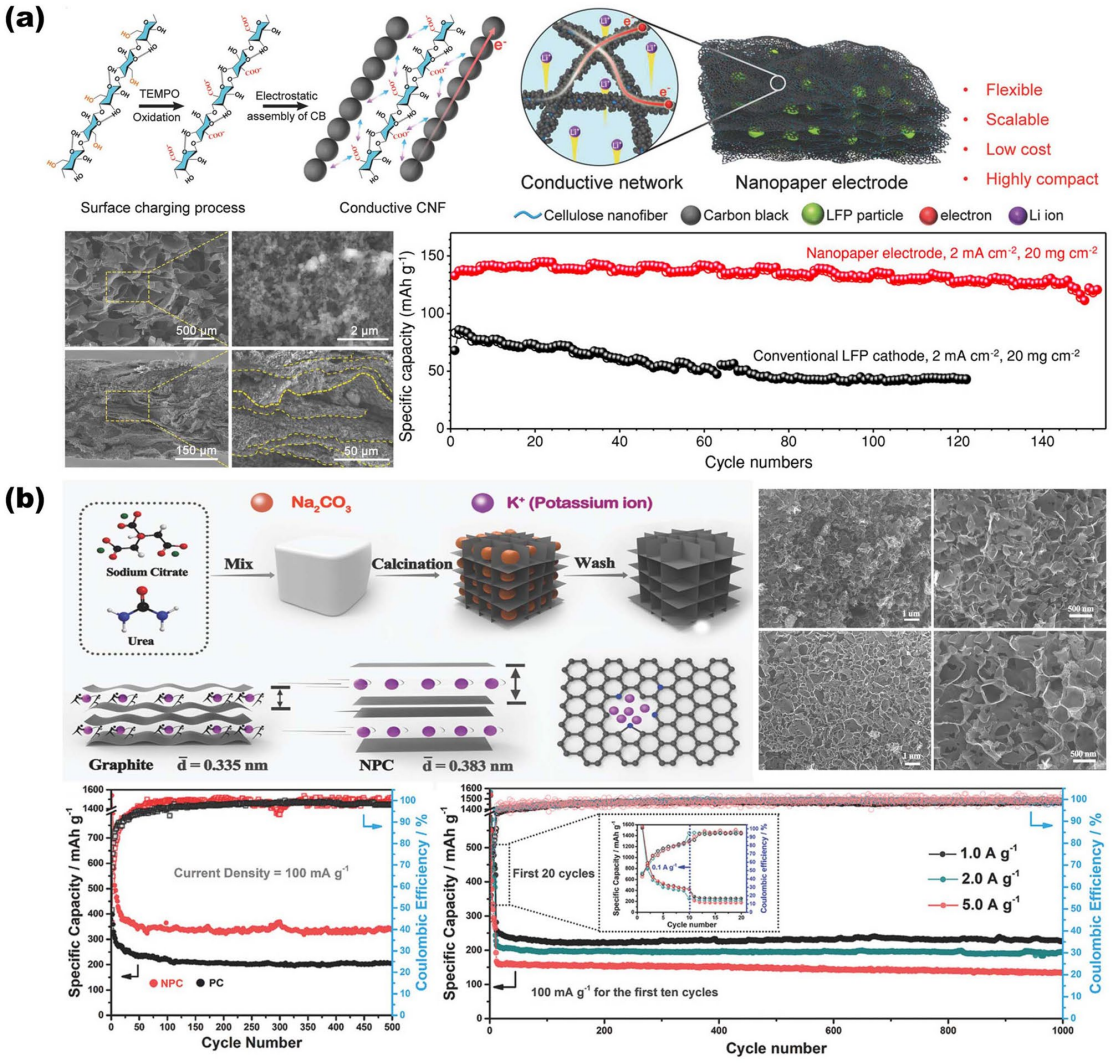
Overview of representative NMS scaffolds for AIBs. **a** Interconnected 3D CNF NMS scaffold with tightly wrapped LFP as close-packed nanopaper electrode for Li-ion batteries. Reproduced with permission from Ref. [120]. Copyright 2018, Wiley‐VCH. **b** Porous carbon synthesized via a self-template technique as both an electrode and a scaffold for K-ion batteries. Reproduced with permission from Ref. [15]. Copyright 2018, Wiley‐VCH

In the past few years, designing NMS scaffolds for AIBs has emerged not only as an attractive strategy to realize enhancement both of power density and rate performance without at the expense of a much lower energy density, but it is also used to ensure sufficient charge–discharge life cycles. Conductive network scaffolds based on one-dimensional nanostructures (such as CNTs, carbon nanofiber, and cellulose nanofiber (CNF)) show significant potential in the field of flexible wearable energy storage devices due to numerous advantages. Case in point, Kuang and coworkers reported a conductive nanofiber network-based CNF scaffold that possesses mechanical robustness and good electrolyte retention, offering decoupled ion and electron transfer pathways to ensure fast charge transfer kinetics of the electrode [38, 42, 86, 87]. As shown in Fig. 7a, after pretreated process of CNF, neutral carbon black particles are assembled onto the surface of negatively charged CNF via spontaneous electrostatic self-assembly technique, forming the versatile scaffold for thick electrodes with compact structure [120]. Attributing to the unique interconnected 3D network with tightly wrapped lithium ion phosphate (LFP) endowed by freeze-drying process, the close-packed nano-paper electrode obtained due to the densification treatment performs high active material mass loading up to 60 mg cm^−2^. Eventually, the Li-LFP batteries deliver high areal capacity and volumetric energy density of 8.8 mAh cm^−2^ and 538 Wh L^−1^, along with better cycling stability and capacity retention (90% after 150 cycles). As for potassium-ion batteries (PIBs), considering the large radius of K^+^ (1.38 vs 1.02 Å of Na^+^ and 0.76 Å of Li^+^), a robust scaffold with a larger space is needed to buffer the huge volume change so as to facilitate the fast K ions insertion–extraction processes during the cycles, preventing the subsequent pulverization of active materials. Compared to graphitic carbon with confined interspace, owing to its larger interlayer spacing, hard carbon is a more suitable candidate as anode material for PIBs to form stage-1 potassium intercalation compounds. Based on this concept, Li and coworkers illustrated a hierarchically nitrogen-doped porous carbon (NPC) synthesized via a self-template technique as both an electrode and a scaffold for K ion insertion–extraction processes (Fig. 7b). It is noteworthy that urea is employed as a nitrogen source in the synthesis process to graft various nitrogen doping groups into the obtained porous carbon matrix, both for enlisting more electronegative to generate a stronger attraction toward ions and for expanding carbon layer space via tuning electronic structure [15]. Finally, the obtained NPC electrode results in a rate capability of 185 mAh g^−1^ at 10.0 A g^−1^ and a high reversible capacity of 342.8 mAh g^−1^ after 500 cycles, which exhibits good enough electrochemical performance as PIB carbonaceous anode.

#### NMS Scaffold Designs for AIMBs

**Fig. 8:**
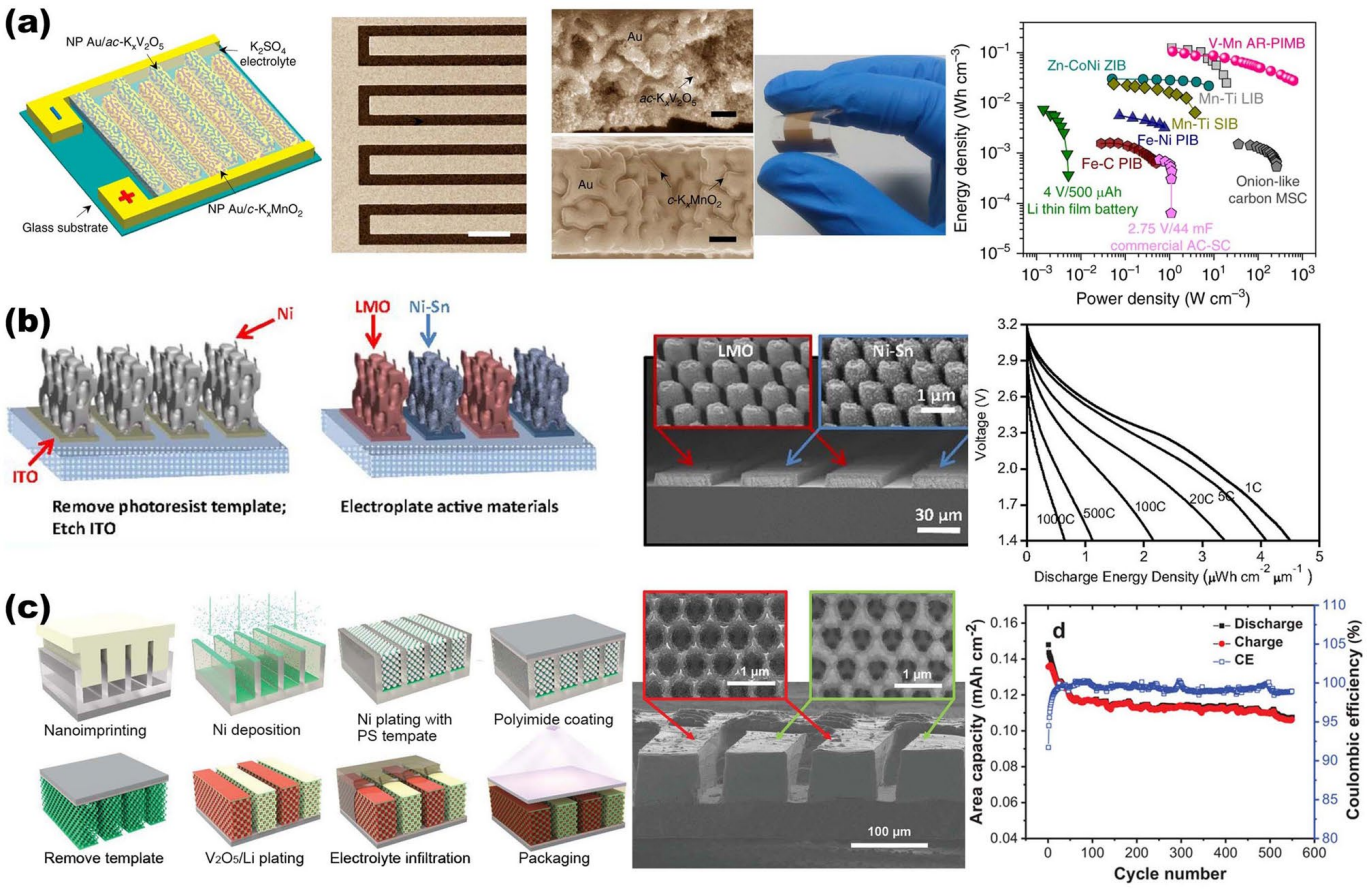
Overview of representative NMS scaffolds for AIMBs. **a** Porous Au simultaneously acts both as micro-current collectors and NMS scaffolds, integrating the c-K_x_MnO_2_ cathode and ac-K_x_V_2_O_5_ anode for Li-ion micro-batteries. Reproduced with permission from Ref. [121]. Copyright 2019, Springer Nature. **b** 3D Ni holographic scaffold, integrating the NiSn anode and LiMnO_2_ cathode for Li-ion micro-batteries. Reproduced with permission [29]. Copyright 2015, National Academy of Sciences. **c** Interconnected Ni porous NMS scaffold, integrating the Li metal anode and V_2_O_5_ cathode for Li-ion micro-batteries. Reproduced with permission [71]. Copyright 2021, Wiley‐VCH

In the era of the Internet of Things (IoT), the requirement for autonomous power supplies from numerous miniaturized electronic devices is becoming more urgent. In order to be compatible with other miniaturized devices, the footprint area of AIMBs is limited within 1 cm^2^ as well (similar to MSCs mentioned in Sect. 4.1.2). Therefore, utilizing NMS scaffolds to construct 3D electrodes for AIMBs has been recognized as a potent way to achieve optimized power density and cycling stability without sacrificing energy density. In detail, despite the enlarged amount of active material loaded, the usage of 3D space in the NMS scaffold ensures a shorter ion transport diffusion length than that of 2D thick electrodes, which is crucial for AIMBs to realize simultaneous enhancement of the energy and power. Over the past few years, there has been a growing realization that tailoring the structures of NMS scaffolds precisely is a key aspect of many of these AIMBs, as it endows the devices with some predictable physical or chemical properties and functions in the context of fast processing technology development. Among various processing techniques, chemically de-alloying is a desirable approach to obtaining interconnected 3D NMS metallic scaffolds [119, 121]. As reported by Li and coworkers, endowed by HNO_3_ de-alloying treatment of interdigital-patterned Ag_75_Au_25_ (at%), the porous Au simultaneously acts both as micro-current collectors and NMS scaffolds, with each microelectrode structure consisting of periodic Au ligaments and nanopore channels (Fig. 8a). When integrating the c-K_*x*_MnO_2_ cathode and ac-K_*x*_V_2_O_5_ anode on Au porous NMS current collectors as full cell, this really realizes Li-ion micro-battery-like capacity as well as supercapacitor-level rate performance and cycle life span [121]. The maximum energy density of the device exhibits ~ 103 mWh cm^−3^ which is 14-fold higher than the Li-film battery (4 V/500 μAh), along with ~ 600 W cm^−3^ power density.

To precisely tailoring the structures of NMS scaffolds to comprehensively optimize the electronic and mechanical performance of AIMBs, a well-known processing technique is photolithography. Over the past two decades, to some extent, the photolithography technique has been the central aspect of intensive investigations and practical applications for manufacturing miniaturized electronic devices. A surge in the development of photolithography has allowed the rapid upscaling of the AIMBs both in design and innovation in the past 5 years. Ning and coworkers demonstrated a strategy that features the synergy between 3D holographic lithography and conventional photolithography, enlisting simultaneous control of both the scaffold structure and spatial arrangement of AIMBs. From an application point of view, this is a compatible strategy for commercial manufacturing. Thanks to recent advances, which have enabled a technological upgrade that enables the complex construction of well-defined periodic NMS porous electrodes using a single incident beam and standard photoresist treatments by varying the beam patterns and exposure parameters. Specifically, by varying the parameters of the holographic lithography beam (for instance, intensity, polarization, and angle), various structures can be realized via photoresist templates for confining the electrodeposition growth behavior of scaffold materials, resulting in customized 3D NMS scaffolds after removing the photoresist templates. As shown in Fig. 8b, following the concept of effective electron and ion transportation pathways, both of interdigitated LiMnO_2_ cathodes and Ni–Sn anodes are based on 3D NMS Ni scaffolds, resulting in supercapacitor-like power for Li-ion micro-batteries (3600 μW cm^−2^ μm^−1^ peak) [29]. In another recent work, Sun and coworkers reported a promising strategy for merging imprint lithography, self-assembly, and electrochemical deposition to obtain an interconnected porous NMS scaffold for high-performance interdigitated AIMBs (Fig. 8c) [71]. Distinguished from the conventional photolithography used above (Fig. 8b), they impress silicon template into melted a poly (methyl methacrylate) (PMMA) and then separated the silicon after cooling to obtain the interdigitated patterns. Moreover, essential steps in the formation of an interconnected porous NMS Ni scaffold are the self-assembly in the trenches of polystyrene (600 nm colloids), Ni electrodeposition from the bottom to fully fill the trenches, and removal the polystyrene and PMMA by toluene. This interconnected NMS Ni current collector possesses inverse opal structure as shown in SEM image (Fig. 8c), which ensures high active volume fraction simultaneously at high-enough mass loading of active materials, sufficient electrolyte penetration, and fast kinetics. After electrodeposition of V_2_O_5_ (cathode) and Li metal (anode), capillary force guided gel electrolyte infilling, and packaging processes, the assembled AIMBs based on NMS Ni scaffold performs high areal energy and power densities of 1.24 J cm^−2^ and 75.5 mW cm^−2^, respectively.

The use of 3D scaffolds is a promising approach for high-performance ion battery electrodes, enlisting high mass loading of active materials, fast electron and ion kinetic transport pathways, and robust mechanical properties. Active materials can be simply formed as thin films on the surface of NMS scaffolds in a conformal manner by infiltration, spraying, or deposition. For network-based 3D scaffolds based on 1D and 2D materials, the active material is simply wrapped into the scaffolds, and then the interconnected network structure becomes a stable support for the active material with good interfacial connectivity without the need for additional binders. For 3D scaffolds, the conformal attachment of the active material becomes extra important. On the one hand, the 3D porous interconnected structures provided by NMS scaffolds offer abundant active sites and excellent transfer kinetics; on the other hand, despite being thin films, the use of 3D NMS scaffolds endows active materials with sufficient mass loading for energy storage due to the utilization of third-dimensional space. Therefore, especially for AIMBs, thin film deposition techniques are more suitable for loading active materials onto NMS scaffolds, including atomic layer deposition (ALD), electrochemical deposition, chemical vapor deposition (CVD), and so forth [18, 46, 86, 103]. In fact, although significant progress has been made in recent years, designing and constructing 3D NMS scaffolds for ideal batteries and AIMBs are still in its infancy. The main technological barrier restraining the practical application of NMS scaffolds for batteries and AIMBs is focusing on rational design and precious manufacturing techniques. Moreover, a deeper understanding of the relationship between electrochemical reactions and electrode structure also plays a crucial role in further pursuit in the realm of batteries, where theoretical simulation and analysis are practically necessary.

### Updated NMS Scaffolds for Anode of Metal Batteries

Given the current progress of metal-ion batteries, the energy storage capacity of which is still challenging to satisfy the increasing demands for high energy density of large-scale energy storage technology. Owing to the fact that the capacity of commercially graphite anode is close to theoretical capacity ceilings, developing new battery system that directly utilizes metals with high theoretical specific capacities (Li: 3860 mAh g^−1^; Na: 1166 mAh g^−1^; K: 685 mAh g^−1^; Zn: 820 mAh g^−1^; Al: 2980 mAh g^−1^; Mg: 2200 mAh g^−1^) and low electrochemical potentials (for instance, Li-metal battery: −3.04 V; Na-metal battery: −2.71 V; K-metal battery: −2.93 V compared with hydrogen electrode) as anode to obtain breakthrough in energy density is an alternative avenue for next-generation high-performance rechargeable batteries [137–142]. However, the stability issue of metal batteries restricts their practical application. This is due to the fact that metal anodes are highly susceptible to dendrite formation produced by uneven metal deposition during long-term charging and discharging, which can pierce the diaphragm and lead the battery to short-circuit [143–145]. Furthermore, preferential dendritic root dissolution increases the danger of dendrite separation from the current collector, resulting in a decrease in Coulombic efficiency during cycling as well as premature battery failure [146, 147]. In order to enhance the energy storage performance of metal batteries, researchers are focusing on discovering methods to overcome the dendrite problem in the anode.

#### Advanced NMS Scaffolds as Current Collector of Metal Anodes

**Fig. 9:**
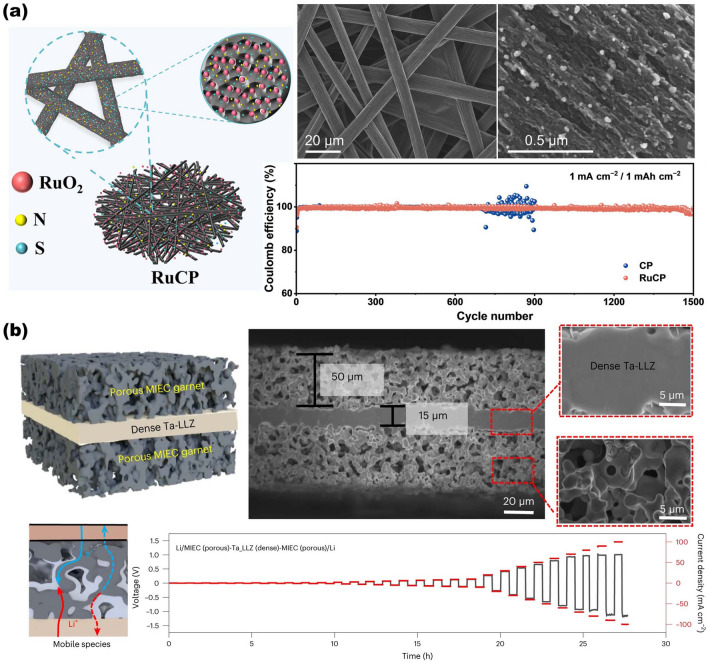
Overview of representative NMS scaffolds as anode current collectors for metal batteries. **a** RuCP scaffolds composited from carbon paper and RuO_2_ nanoparticles for Na-CO_2_ battery anodes. Reproduced with permission from Ref. [150]. Copyright 2023, Wiley–VCH. **b** Single-phase MIEC as an anode collector for Li-metal batteries. Reproduced with permission from Ref. [63]. Copyright 2023, Springer Nature

Due to the uneven electric field and ion flux on the surface of conventional planar electrodes, the instability of the metal anode during the plating-stripping process usually results in dendrites [148]. The construction of porous conductive substrates based on NMS scaffolds can dissipate the inhomogeneous local current density and ion flux, effectively avoiding the generation of dendrites [149]. At the same time, the interconnection network effectively mitigates the volume change of the metal material during repeated deposition/stripping processes and stabilizes the electrode electrochemical performance. Moreover, the sufficient internal space of the porous structure can fully accommodate the metal deposition while ensuring smooth ion transport channels, which assists in managing the metal deposition effect. Lei's group has synthesized composite NMS scaffolds that can serve as Na metal anode hosts by electrochemically dispersing RuO_2_ nanoparticles homogeneously on the fiber surface of carbon paper (RuCP), as shown in Fig. 9a [150]. The interwoven carbon nanofibers in the RuCP scaffolds provide an electron-conducting network. While the RuO_2_ nanoparticles exhibit a strong affinity for Na ions, which promotes the ability of the Na metal to uniformly wrap around the surface of the scaffold and fill the voids, significantly reducing the surface energy of carbon paper. SEM images of the scaffolds before and after Na metal deposition indicated that Na metal evenly coated the RuCP scaffolds and produced a dense and smooth surface following a Na deposition procedure of 20 mAh cm^−2^. The electrode overpotential was just 13 mV at 1 mA cm^−2^, allowing for lengthy periods of steady dendrite-free operation (1500 cycles). Alexander et al. created and employed a single-phase mixed ion- and electron-conducting garnet (MIEC) NMS scaffold in Li-metal batteries [63]. As shown in Fig. 9b, the MIEC has approximately conductivity for both lithium ions and electrons. Combined with the continuous and porous structure, it is capable of uniformly distributing potential across the whole surface throughout the charging and discharging process. It is an effective strategy to alleviate the tensions on the solid electrolyte surface, hence decreasing localized hot spots that can contribute to dendrite formation. The authors demonstrate that the critical current in a sandwich symmetric structure comprised of a MIEC support and a garnet solid electrolyte can achieve an unprecedented 100 mA cm^−2^ without dendritic short-circuiting. In addition, the hybrid solid-state battery also exhibits a long cycle life (350 and 500 cycles at 1.15 and 2.3 mA cm^−2^ current densities, with a cathode area capacity of 2.3 mAh cm^−2^).

**Fig. 10:**
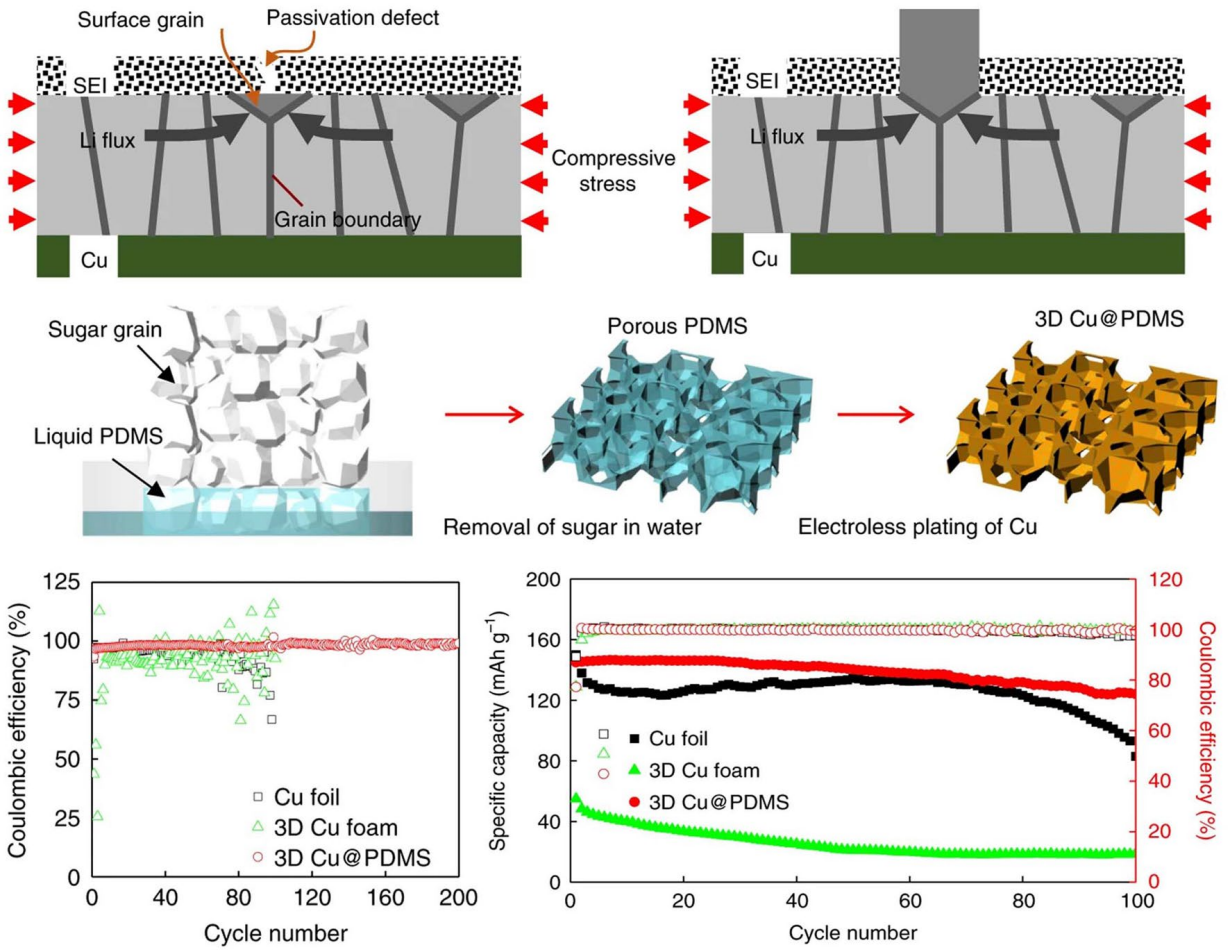
Modeling of internal stress-driven dendrite generation and design of flexible 3D Cu@PDMS scaffolds for Li-metal anode collectors. Reproduced with permission from Ref. [35]. Copyright 2018, Springer Nature

In addition to the impacts of electric field and ion flux on dendrites, internal stresses during metal deposition are recognized as an essential aspect in dendrite existence, as demonstrated by theoretical model of Jiang's group [35]. The stress-driven metal diffusion flux transfers and deposits metal ions at the interface of the solid electrolyte interface (SEI) bottom layer during Li deposition. When the SEI layer is defective at this point, Li dendrites form here. The mechanical property of the continuous porous NMS scaffold serves to counteract the internal stresses in the material, thus mitigating the generation of dendrites at this level. Based on this concept, the group reports a 3D porous Cu collector supported by a soft PDMS substrate (shown in Fig. 10). It is shown that the internal stress during Li metal deposition leads to folds in the soft collector and gradually transforms from a 1D to a 2D wrinkling pattern. The wrinkling releases the internal stresses during Li plating and suppresses the emergence of dendrites. The half-cells fitted with soft Cu collectors have a Coulombic efficiency of more than 98% at a current density of 1 mA cm^−2^ and more than 200 cycles.

#### Advanced NMS Scaffolds as SEI Layer of Metal Anodes

**Fig. 11:**
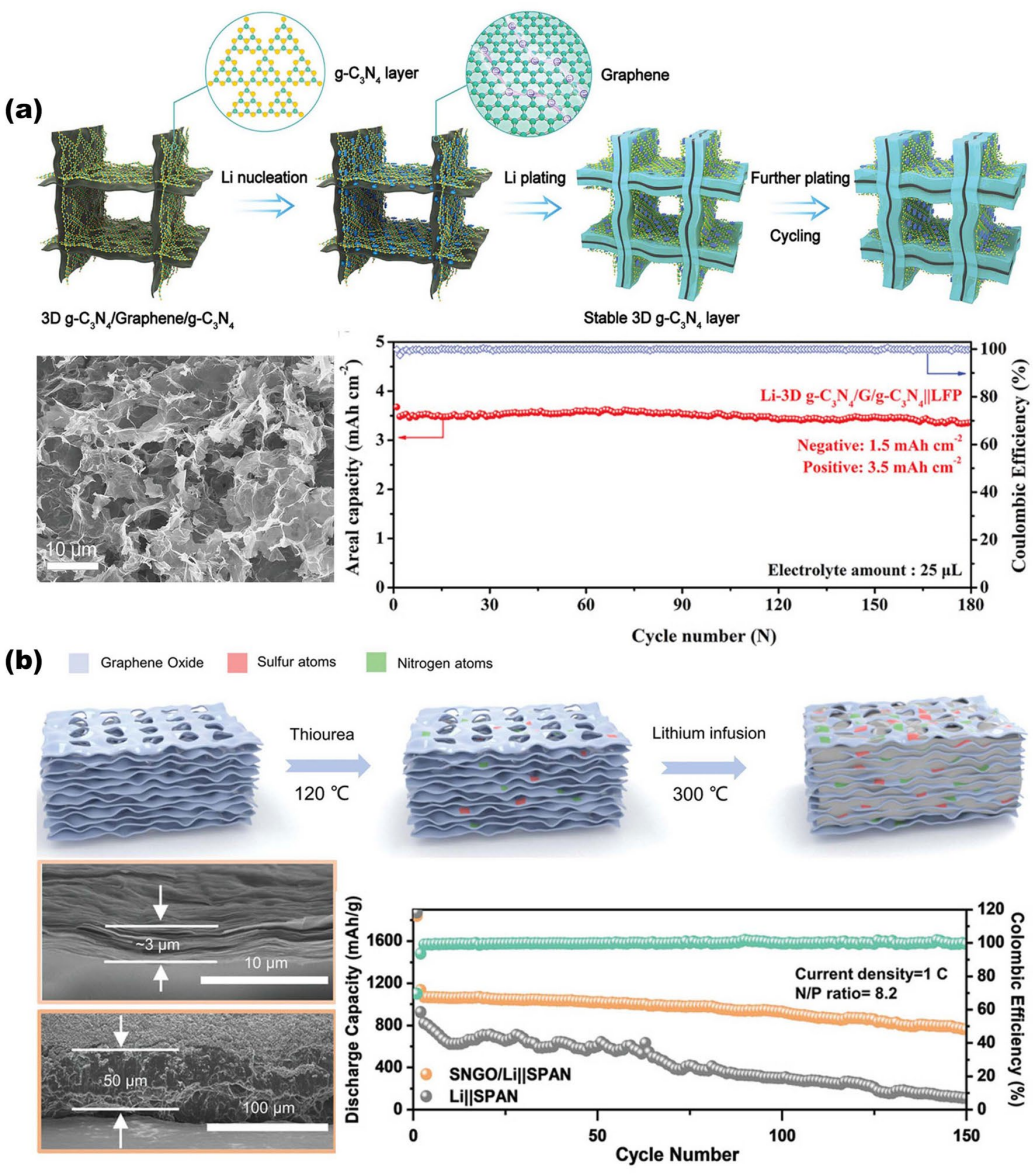
Overview of the SEI layer for metal battery anodes based on NMS scaffolds. **a** 3D sandwich structure composite scaffold composed of g-C_3_N_4_/graphene/g-C_3_N_4_ for Li metal battery anode. Reproduced with permission from Ref. [40]. Copyright 2021, Wiley–VCH. **b** The SNGO framework possesses Li_3_N-Li_2_S SEI layers for improving Li metal anode performance. Reproduced with permission from Ref. [20]. Copyright 2022, Wiley–VCH

Another important aspect that affects the stability of the metal cell anode is SEI layer. As known, the SEI layer refers to a layer of film formed between electrolyte and electrode material that can prevent side reactions between electrode material and electrolyte, so as to improve the performance and safety of the battery. In practice, however, the metal battery anode will cause the SEI layer to break/reorganize continuously under the huge volume change during the reaction process, which will lead to an increase in lithium ion diffusion resistance at the interface and an elevation of voltage polarization. Therefore, utilizing NMS scaffolds as artificial SEI layers is another appealing strategy to enhance electrode stabilization. Generally speaking, the artificial SEI layer should fulfill the following requirements: (1) excellent chemical and electrochemical stability, with electronic insulation to prevent the successive depletion of the electrolyte and the metal; (2) good mechanical strength and flexibility, which can inhibit the huge volume change and dendrite growth of the anode in the process of charging and discharging; and (3) the ability for fast and uniform cross-layer transport of metal ions. The properties of NMS scaffolds can fully satisfy the demands of an artificial SEI layer and realize the effective protection of metal electrodes. As shown in Fig. 11a, Zhai et al. designed and prepared insulator/metal/insulator 3D sandwich structure composite scaffold consisting of g-C_3_N_4_/graphene/g-C_3_N_4_ for Li-metal battery anodes by a combination of 3D self-assembly and in situ calcination [40]. Among them, g-C_3_N_4_ serves as an artificial SEI layer with insulating properties that inhibit Li metal deposition on its surface, while its unique structured nanopores allow Li^+^ penetration to reach the interior and insulate the electrolyte. Since g-C_3_N_4_ is connected to graphene by van der Waals forces, there is enough internal gap to allow Li deposition on the graphene surface. The interconnected structure and mechanical properties provided by the graphene network greatly lower the local current density and volume change during lithium deposition and exfoliation. And the amorphous morphology of g-C_3_N_4_ results in more homogenous and protective characteristics due to the lack of brittle grain boundaries. The synergistic effect of g-C_3_N_4_ with graphene enables the 3D scaffold anode to maintain high Coulombic efficiency (99.89%) and reliable long-term cycling (180 cycles) under high cathode capacity (3.5 mAh cm^−2^) and lack of electrolyte (25 μL per cell). Otherwise, the NMS scaffold can also assist in the formation of the SEI layer and stabilize its function. Besides, in comparison, the ordered 3D structure not only has better mechanical properties and stability but also can better reveal the structure–property relationship through theoretical simulation, so as to investigate the influence mechanism and provide guidance for structural design. Ni and colleagues assembled ordered micropore-structured graphene oxide scaffolds doped with S and N atoms (SNGO) (Fig. 11b) [20]. Following a molten lithium infusion process, stable Li_3_N and Li_2_S can be formed on the film at the same time as the anode SEI layer to realize the rapid transmission of Li ions. The lithiophilic properties of N and S functional groups can promote the uniform deposition of Li ions. Moreover, the graphene oxide scaffolds provide sufficient accommodation space while alleviating the volume expansion caused by Li deposition, thus improving the strength and toughness of the SEI layer and avoiding the occurrence of fracture. COMSOL simulations also confirmed that the 3D-printed ordered microchannels further shorten the Li ion transport pathways and uniformly disperse the internal electric field, heat, and stress distribution, preventing dendrite formation. The measured results of the assembled Li–S full battery show that the Li anode composed of SNGO has a stable cycling performance with a reversible discharge capacity of up to 861.7 mAh g^−1^ after 250 charge/discharge cycles at 2 C rate. This presents a new concept for the design of artificial SEI layer. In addition, some other typical examples of NMS used for metal battery anodes are summarized in Table 3.

**Table 3:** Electrochemical performance of metal batteries anode based on NMS scaffolds

Functionality	Anode metal	Scaffolds	Main techniques	Half cells (current density (mA cm^−2^), areal capacity (mAh cm^−2^), cycle umber)	Symmetric cell (current density (mA cm^−2^), areal capacity (mAh cm^−2^), life span (h))	Full cells (cathode, rate performance, and life span)	Refs
Current collector	Li	MIEC garnet	Scalable tape-casting process	–	1, 1, 50 & 5, 5, 50	NMC622, 1C, 500 cycles	[63]
	Li	Cu-coated carbon fabrics	Polymer-assisted metal deposition	1, 3.5, 90	1, 2, 500	NSHG/S_8_/NiCF, 1 (mA cm^−2^), 260 cycles	[151]
	Li	Cu/Ni core–shell network	Hydrothermal and sintering	2, 1, 100	3, 1, 208.3	LiCoO_2_, 1C, 200 cycles	[152]
	Li	3D Cu&CuAu_x_ matrix	High energy heavy iontracking	–	1, 1, 2160	LiFePO_4_,10 C, 200 cycles	[153]
	Li	3D porous CuZn	Dealloying	0.5, 0.5, > 800	0.5,1, > 500	NCM811, 1 C, 500 cycles	[154]
	Li	3D printed porous Cu	3D printing	1, 5, > 450	1, 2.5, 450	–	[155]
	Li	P-Cu@Cu_6_Sn_5_	Electroless plating	1, 1, 325	1, 1, > 1000	LiFePO_4_, 1 C, 600 cycles	[156]
	Zn	3D Ti_3_C_2_T_x_ nanosheets	Freeze-drying	5, –, 150	1, 1, 1500	VO_2_, 5 A g-1, 1000 cycles	[43]
	Zn	3D Ti-TiO_2_	Template-free electrodeposition and vapor dealloying	5, 1, 120	1, 1, 2000	S-MXene@MnO_2_, 5 (A g^−1^), 500 cycles	[157]
	Zn	3DCEP-MXene	3D cold-trap environment printing	1, -, 450	0.25, 0.1, 1400	3DCEP-MXene/Co-MnHCF, 0.9 C, 1600 cycles	[158]
	Na	3DHS with Mg clusters	Solution mixed and carbonization	–	0.5, 1, 450	FeS_2_, 0.5, 50 cycles	[159]
	Na	3D Zn@Al	Magnetron sputtering	0.5, 0.5, 1200	2, 1, 1500	NVP, 5 C, 2000 cycles	[72]
	Na	SnO_2_-CNFs	Carbonizing PAN nanofibers and magnetron sputtering	3, 3, 1500	1, 1, 3000	NVP@C@CNTs, 1 C, 130 cycles	[41]
	K	Co/NOC/CNM	Solution mixed annealing	0.5, 0.5, 110	1, 1, 1000	PTCDA, 10 C, 100 cycles	[160]
Current collector & SEI layer	Li	g-C_3_N_4_/graphene/g-C_3_N_4_	3D self-assembly and in-situ calcination	1, 1, 500	–	LiFePO_4_, 0.3 C, 180 cycles	[40]
	Li	3D Cu_2_S NWs–Cu foam	Anodizing and sulfuration	1, 1, 500	–	LiFePO_4_, 0.5 C, 300 cycles	[161]
SEI layer	Li	SNGO	Hydrothermal and 3D printing	–	1, 1, 450	NCM811, 0.5 C, 100 cycles	[20]
	Li	NiFx@NF	One-step fluorination	1, 1, 450	1, 1, 1300	LiFePO_4_, 2 C, 500 cycles	[162]
	Li	ZnO–PAN–ZnO skeletons	Electrostatic spinning	0.5, 1, 120	3, 1, 200	LiFePO_4_, 1 C, 200 cycles	[163]
	Li	LiF-rich 3DSF	Toroidal magnetic field	1, 1, 100	1, 1, 1700	LiFePO_4_, 1 C, 150 cycles	[164]

MIEC, mixed ion- and electron-conducting; NMC622, LiNi_0.6_Mn_0.2_Co_0.2_O_2_; NSHG/S8/NiCF, graphene/sulfur mixture on Ni-coated carbon fabric; NCM811, LiNi_0.8_Co_0.1_Mn_0.1_O_2_; S-MXene, S-doped MXene; 3DCEP, 3D cold-trap environment printing; 3DHS, 3D hierarchical structure; NVP, Na_3_V_2_(PO_4_)_3_; NOC, N, O codoped carbon; CNM, carbonaceous nanofiber matrix; PTCDA, perylene-3, 4, 9, 10-tetracarboxylic dianhydride; SNGO, Porous graphene oxide films are doped with sulfur and nitrogen; NF, Ni foam; 3DSF, three-dimensional SEI framework

As mentioned before, for metal battery anodes, NMS scaffold not only improves electron/ion conductivity but also modulates the electric field density and ionic flux on the anode surface, preventing dendrite formation and significantly improving electrode stability. This 3D open structure provides an ideal strategy for the development of metal battery anodes with high energy density and extended cycle life. It is worth mentioning, however, that when utilized as a collector structure, NMS scaffold undoubtedly adds weight to the metal anode while occupying a certain load volume. Constructing NMS scaffolds with hollow structures and high porosity can lessen this influence, but it also affects mechanical characteristics; therefore, the two must be balanced. Furthermore, the relationship between scaffold size and batteries performance when used as SEI layer is not clear. Hole size, porosity, arrangement, and scaffold thickness are all important influencing factors. In addition to experimental studies, in-depth analyses and predictions need to be combined with theoretical calculations, simulations, and machine learning to assist in accurate design.

### Updated NMS Scaffolds for Cathode of Metal-Air Batteries

MABs, a special branch of metal batteries, which are powered by the oxidation of metals and the reduction of oxygen, have emerged as an ideal candidate for the next generation of energy storage devices. Since its cathodic active substance, oxygen, is obtained from the outside atmosphere via the cathode structure rather than stored inside the battery, it is in principle virtually unlimited. This allows MABs to achieve theoretical energy densities tens of times higher than those of conventional metal-ion batteries [165]; for instance, Li-air batteries can achieve an impressive theoretical energy density of 11,429 Wh kg^−1^ and a high specific capacity of 386 mAh g^−1^ (based on the anode Li metal mass) [166], whereas high theoretical energy densities of 1350 Wh kg^−1^ can be obtained for Zn-air batteries as well (based on the anode Zn metal mass) [167]. For typical MABs, the vital oxygen evolution reaction (OER) and oxygen reduction reaction (ORR) during the charging and discharging processes both occur in the contact reaction region between the cathode and other parts (electrolyte, air). Therefore, the air cathode not only provides a reaction site for OER and ORR, but also has to rapidly transport and diffuse the metal ions and gas molecules to the designated interface, which poses higher requirements for the design of the air cathode. Besides, the reaction kinetics of OER and ORR in the air electrode are slower compared with the metal anode reaction. The discharge products generated by the ORR process in non-aqueous batteries tend to accumulate on the electrodes to reduce the efficiency of further reactions [168, 169]. And the air electrode in aqueous electrolyte batteries has functional problems in the transportation of ionic species, leading to lower conversion rates of electrochemical reactions in alkaline aqueous electrolytes and accelerated decay of the battery capacity [170, 171]. These definitely limit the performance of the battery even more. Consequently, precise design and optimization of air cathode structures are essential to maximize energy storage efficiency and prolong the stability of MAB.

The unique interconnected porous structure of the NMS scaffolds is fully utilized and is ideal to handle the numerous challenges faced by MABs cathodes. (1) The porous structure of NMS scaffolds can expose more active sites and provides\stable three-phase reaction interface [172]; (2) Continuous porous channels not only provide high-speed conduction paths for gas molecules and metal ions, but also further regulate the size and morphology of reaction products to enhance the rate performance [173, 174]; (3) The sufficient internal space of the porous structure can alleviate the accumulation of products and ensure the adequacy and stability of the whole discharge process [175]; (4) The suitable NMS scaffold improves the stability and cycle life of the air cathode, as well as catalyzes and facilitates the ORR/OER process to further improve the performance of the MABs. Overall, porous NMS scaffolds containing abundant and continuous diffusion pathways can provide stable three-phase reaction interfaces with outstanding mass transfer efficiencies for batteries in a sustained manner, leading the way to maximize the performance of MABs. Table 4 summarizes several performance comparisons of NMS scaffolds applied to MABs cathodes in recent years.

**Table 4:** Electrochemical performance of MABs cathode based on NMS scaffolds

Battery type	Scaffolds	Main techniques	Electrolyte	Voltage hysteresis, applied current	Peak power density	Discharge capacity	Cyclability	Refs
Li–O_2_	Graphene oxide aerogel/NiCo_2_O_4_	Electrostatic assembly and freeze-drying	0.1 M LiTFSI in DOL/DME (1:1) with 1% LiNO_3_	≈1 V, 0.1 (mA cm^−2^)		3398.4 (mAh g^−1^)	2000 h (400 cycles) at 0.1 (mA cm^−2^) with a cut-off capacity of 0.25 (mAh cm^−2^)	[69]
Li–O_2_	Carbon frameworks/Co	Extrusion-based 3D printing	0.5 M LiClO_4_-DMSO	–	–	798 (Wh kg^−1^)	165 h at a limited capacity of 1 (mA h) and 0.1 (mA cm^−2^)	[176]
Li–O_2_	Porous black TiO_2_	Hydrogen reduction	1 M LiN(SO_2_CF_3_)_2_ in TFGDMF and 0.05 M LiI	0.37 V at 500 (mAh g^−1^)		7500 (mAh g^−1^)	≈340 cycle at 500 (mA g^−1^) and 1000 (mAh g^−1^)	[34]
Li–O_2_	Tri-pathway carbon nanotube/Ru	Chemical delignification and coating process	111 μL cm-2 of 1 m LiTFSI/TEGDME	0.85 V at 100 (mA g^−1^)		7300 (mAh g^−1^)	220 cycles with 1000 (mAh g^−1^) at 200 (mA g^−1^)	[177]
Li–O_2_	Ag/NiO-Fe_2_O_3_/Ag	Thermal treatment	1 M LiCF_3_SO_3_/TEGDME	1.24 V, 100 (mA g^−1^)		5138 (mAh g^−1^)	180 cycles at 300 (mA g^−1^) with a cut-off specific capacity of 1000 (mAh g^−1^)	[37]
Li–O_2_	Au/Cu@FCu	Hydrothermal and reduction methods	1 M LiTFSI in (DOL)/DME (1:1, w/w) with 0.1 M LiNO_3_	0.64 V, 100 (mA g^−1^)		27,270 (mAh g^−1^)	220 cycles with 100 mA g^−1^ at a fixed capacity of 500 (mAh g^−1^)	[178]
Li–O_2_	NiCo_2_O_4_/CNFs	Hydrothermal method	1 M LiTFSI/TEGDME	≈1.7 V, 100 (mA g^−1^)		4179 (mAh g^−1^)	350 cycles at 200 (mA g^−1^) with a cut-off specific capacity of 1000 (mAh g^−1^)	[179]
Li–O_2_	Porous hGO meshes	3D printing	0.1 LiTFSI in DMSO	< 1 V, 0.1 (mA cm^−2^)	–	13.3 (mAh cm^−2^) or ≈3879 (mAh g^−1^)	13 cycles at 0.1 (mA cm^−2^)	[21]
Zn–air	N-doped hierarchically porous carbon plates	Enzyme treatment and subsequent pyrolysis with NH_4_Cl	6 M KOH and 0.2 m zinc acetate	≈0.7 V, 10 (mA cm^−2^)	192.7 (mW cm^−2^)	801 (mAh g^−1^)	110 h at 10 (mA cm^−2^)	[36]
Zn–air	Carbon nanotube network	Oxygen-induced electron density modulation	6 M KOH and 0.2 M zinc acetate	–	130.5 (mW cm^−2^)	–	340 h GCD test at 5 (mA cm^−2^)	[180]
Zn–air	Fe_8_Co_0.2_-NC-800	Pyrolysis of core–shell microspheres	6 M K(OH) and 0.2 M Zn(CH_3_COO)_2_	–	124.9 (mW cm^−2^)	704 (mAh g_Zn_^−1^)	311 h at 5 (mA cm^−2^) with discharge for 5 min and charge for 5 min	[181]
Zn–air	GH-BGQD	One-step hydrothermal route	2.0 M KOH	–	112 (mW cm^−2^)	687 (mAh g^−1^)	300 cycles at a current density of 5 (mA cm^−2^)	[182]
Zn–air	N-droped carbon nanosheets/Co	Salt-template method	6 M KOH	0.63 V	≈194 (mW cm^−2^)	≈690 (mAh g_Zn_^−1^)	20 h at 5 (mA cm^−2^)	[183]
Al–air	3D carbon	Polyaniline-assisted template method	0.1 M KOH	–	130.5 (mW cm^−2^)	–	discharge under 1.27 V for 30 h	[184]
Al–air	Defect-rich MnO_2_ NWs networks	Ar plasma approach	4 M NaOH	–	159 (mW cm^−2^)	–	6 h at a current density of 100 (mA cm^−2^)	[185]
Al–air	N-doped carbon nanosheets	Thermally converted	4 M KOH	–	130 (mW cm^−2^)	552 (mAh g^−1^)	11 h at a current density of 5 (mA cm^−2^)	[186]
Mg–air	Co/nitrogen-doped CNTs	Hydrothermal method and calcination	6 M NaOH	–	58.6 (mW cm^−2^)	–	5000 s at current density of 10 (mA cm^−2^)	[187]
Mg–air	Amorphous-MnO_2_/CNTs-OH	Direct reaction method and heat-treated	15 wt% NaCl	–	79.2 (mW cm^−2^)	–	15 h at a current density of 50 (mA cm^−2^)	[188]

LiTFSI, Lithium bis(trifluoromethylsulfonyl)imide; DOL, 1,3-dioxolan; DME, 1,2-dimethoxyethan; DMSO, dimethyl sulfoxide; TEGDME, tetraethylene glycol dimethylether; GH-BGQD, B-doped graphene quantum dots anchored on a graphene hydrogel; NWs, nanowires; CNTs, carbon nanotubes

**Fig. 12:**
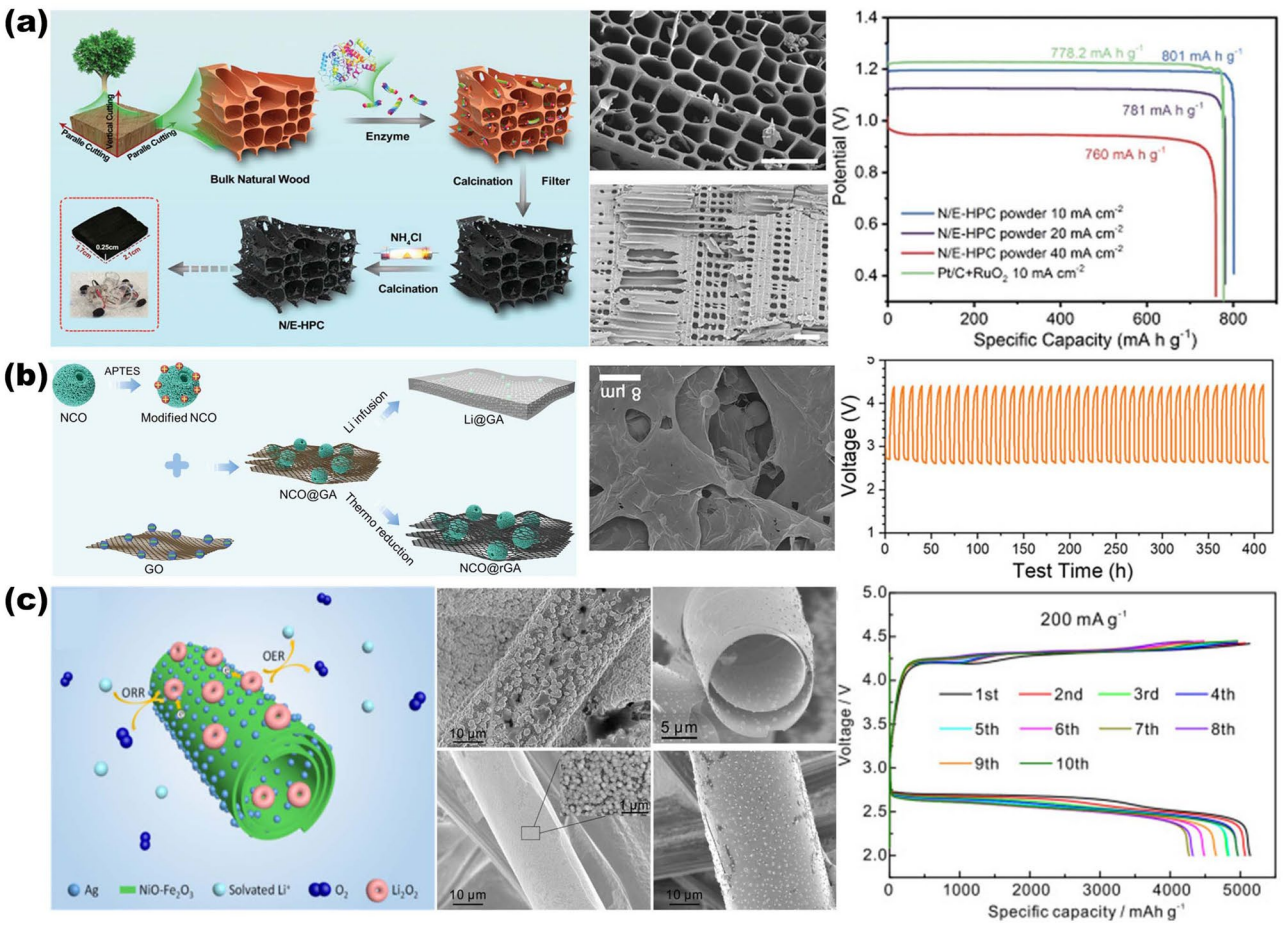
**a** Preparation of N-doped carbon porous scaffolds from virgin wood for Zn-air battery cathodes. Reproduced with permission from Ref. [36]. Copyright 2019, Wiley–VCH. **b** NiCo_2_O_4_ microspheres embedded into 3D graphene oxide aerogel (NCO@rGA) for Li–O2 battery air electrode. Reproduced with permission from Ref. [69]. Copyright 2020, Wiley–VCH. **c** Ag/NiO-Fe_2_O_3_/Ag (Ag/NFO/Ag) hybridized micrometer tube porous structure for Li–O2 air cathode. Reproduced with permission from Ref. [37]. Copyright 2018, Elsevier

Porous carbon materials were selected as popular materials for air cathode NMS scaffolds due to their high specific surface area, large porosity, and light weight. Peng et al. designed N-doped carbon scaffolds with plentiful holes by creating a high number of micropores from virgin wood as a raw material via facile pyrolysis and catalytic cellulose hydrolysis. It can be utilized directly as the cathode of Zn-air batteries (as exhibited in Fig. 12a) [36]. The lignocarbon material with a large specific surface area completely exposes the active sites while maintaining a mechanically robust crosslinked network of scaffold structures. Furthermore, a significant number of OER-active pyridine N sites were inserted into the carbon skeleton, which demonstrated good ORR and OER catalytic activities. This scaffold-based reversible Zn-air battery attained a current capacity of 801 mAh g^−1^ and exhibited no performance loss after 110 h of continuous operation. Other carbon materials, including as carbon cloth, carbon black, and Ketjenblack, can also form a structural network and be utilized as air cathodes. However, due to weak OER catalytic activity, they are prone to corrosion/oxidation issues at high charge potentials, leading to impaired battery performance. Thus, researchers combined some high-catalytic activity non-carbon materials with carbon materials to avoid this problem. Ma et al. used electrostatic assembly and freeze-drying techniques to embed NCO microspheres into 3D graphene oxide aerogel (NCO@rGA) [69]. As shown in Fig. 12b, this composite scaffold was built and employed as the structural backbone of both the anode and cathode of a Li–O_2_ battery. The combined Li–O2 batteries were able to achieve more than 400 highly reversible discharge/charge cycles, and exhibiting good long-term cycling stability and mechanical flexibility. The analysis reveals that the NCO@rGA composite scaffold in the anode aids in dispersing the local current density and mitigating the volume change of Li metal during charging and discharging. When employed as a cathode, it not only offers a loose conductive network, but its high OER activity may greatly minimize the charging over-point site. As a result, the service life of the battery is effectively prolonged. Building an air cathode with carbon-free scaffolds can eliminate the problem of battery failure caused by the parasitic interaction between carbon materials and oxidation products. Numerous NMS scaffolds consisting of metals, oxides, nitrides, and carbides have been developed for advancement of MABs. Lu et al. prepared Ag/NiO-Fe_2_O_3_/Ag (Ag/NFO/Ag) hybridized microtube network cathodes by electron beam vapor deposition and heat treatment processes (shown in Fig. 12c) [37]. The main feature is that the material precursors are deposited in the order of metal–oxide–metal in advance and then automatically curled into tubes due to the intrinsic strain by heat treatment, while the surface metal shrinks into nanoparticles attached to the surface under the effect of surface tension. The strain release effect of the heat treatment effectively improves the toughness and structural stability of the micrometer tubes. In addition to its superior catalytic activity, Ag/NFO/Ag provides an internal space that regulates the O_2_ diffusion and electrolyte conduction efficiency during the charging and discharging reactions. The constructed Li–O2 cells exhibit enhanced electrocatalytic activities, including low overvoltage, high capacity retention, and good cycling stability.

**Fig. 13:**
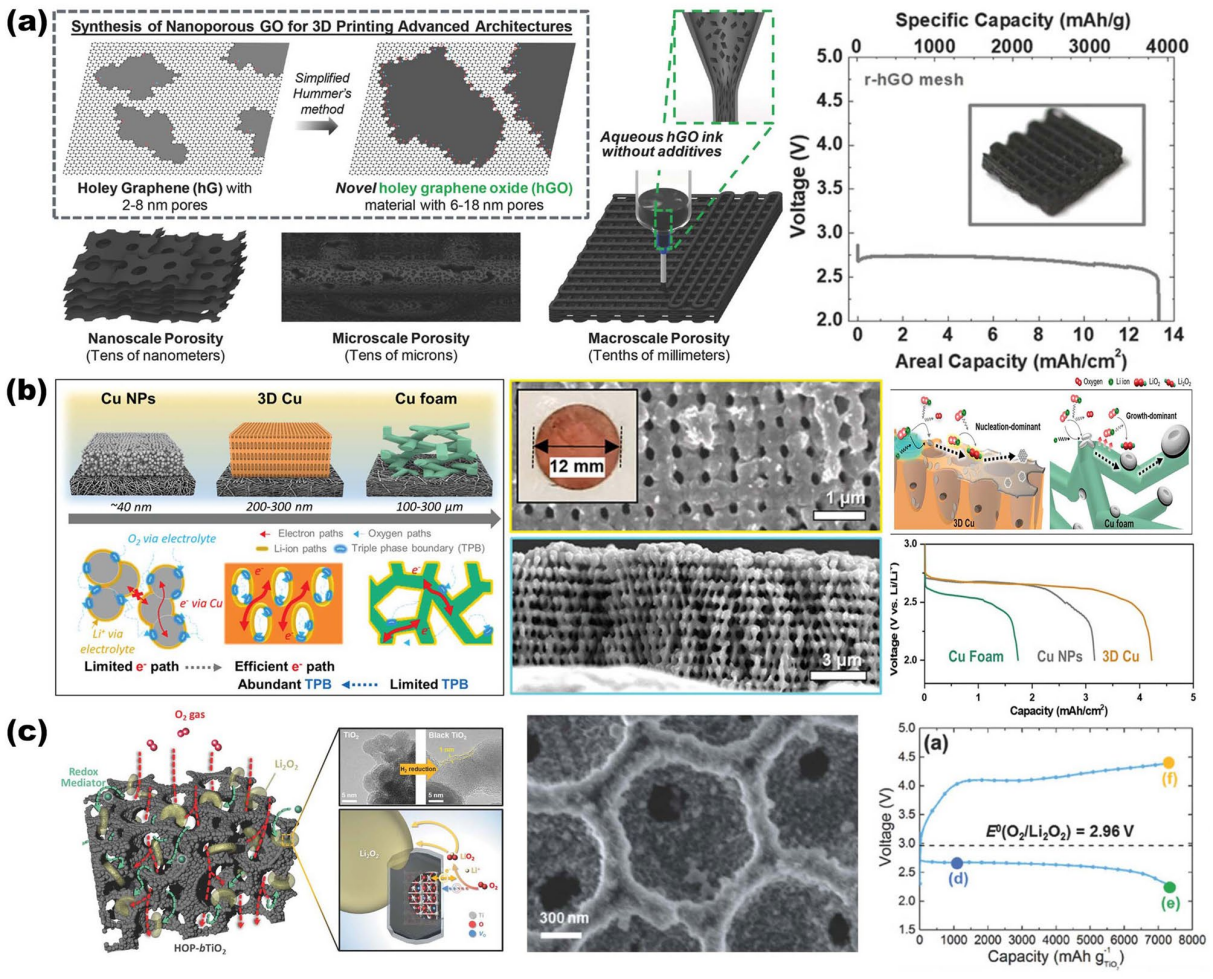
**a** 3D printed graphene oxide hierarchical porous structure scaffolds for air cathodes. Reproduced with permission from Ref. [21]. Copyright 2018, Wiley–VCH. **b** Photolithographically prepared 3D ordered porous Cu electrodes for Li–O2 battery cathodes. Reproduced with permission from Ref. [44]. Copyright 2023, Wiley–VCH. **c** Ordered macro/mesoporous structure of anoxic black TiO_2_ scaffolds and its use as air cathode for growth kinetic modulation of Li_2_O_2_. Reproduced with permission from Ref. [34]. Copyright 2017, Wiley–VCH

Not only does the material selection affect the energy storage capacity and stability of the battery, but the stable three-reaction interface offered by the conductive permeation network is critical to assuring charging and discharging performance. However, the single pore size in the scaffold structure makes it necessary for the electrolyte and air to share the same voids for ion and gas conduction. In reality, this causes pore competition, resulting in unequal responses and low energy density at the interface [9]. To achieve a dual-channel electrode, hierarchical porous structure may efficiently decouple both air and electrolyte channels. Ideally, small pores with high capillary pressure are used to absorb and transport electrolyte, whereas wide pores are designed for gas diffusion. Furthermore, the large area of wide pores can provide sufficient morphological options for discharge products to avoid their entering the micropores and aggregating. To develop hierarchical porous scaffolds and optimize battery performance, it is critical to tune the porous material size and assembly behavior [189]. As shown in Fig. 13a, Lacey et al. prepared aqueous carbon-based ink using oxidized porous graphene and designed NMS scaffolds with hierarchical porous structures using 3D printing technology [21]. The porosity of the freestanding porous graphene oxide networks is classified into three levels: nanoscale (4–5 nm), microscale (tens of microns), and macroscale (< 500 μm square pores). The authors pointed out that this hierarchical porous structure not only fully exposes the active sites, but also provides distinct transport pathways for oxygen and electrolyte, enhancing ORR and OER reactions even further. The 3D-printed network scaffolds outperformed the 2D structured electrodes in terms of battery performance (area capacity of 13.3 mAh cm^−2^, a 63-fold increase). This work demonstrates that hierarchical porous scaffolds are an effective way to improve the performance of next-generation energy storage devices. Furthermore, the highly ordered scaffold structure is practically helpful to understand and study the diffusive flux of air/charge/ions. Based on the spatially aligned pore structure of the 3D patterned cathode, as shown in Fig. 13b, Hyun et al. proved that the homogeneity of the scaffold has important effect in Li–O2 batteries [44]. The highly ordered porous structure with mass transfer path induction leads to higher utilization of the entire air electrode. Meanwhile, the periodic geometrical characteristics of the homogeneous three-reaction interface play an important role in controlling the growth kinetics of the discharge products, which is conducive to the realization of high-performance cells with low overpotentials and high round-trip efficiencies. It is adequate to affect the morphological evolution process of the discharge products because structural surface engineering may effectively regulate the reaction barrier, electronic conductivity, and reaction surface area of the porous cathode. It is also directly related to the charging behavior of the battery. Oxygen-deficient black TiO_2_ scaffolds with an ordered macro-/mesoporous structure (shown in Fig. 13c) were prepared via a simple hydrogen reduction method by Kang and his colleagues [34]. It proved that the highly ordered structure directs Li_2_O_2_ to form ring-like and independent regular particles, which guarantees that Li_2_O_2_ exposes most of its outer surface directly to the electrolyte. In addition, diffusion channels and voids can be maintained at all times so that the electrodes remain open structures. As a result, massive Li_2_O_2_ toroids (300 nm) produced in solution may be effectively charged with soluble catalysts to achieve low polarization voltages (0.37 V).

According to the above, NMS porous scaffolds play an important role in the advanced cathode of MABs. On the one hand, the self-supported continuous porous structure of NMS scaffolds can effectively enhance the transportation efficiency of electrolytes, reactive electrons, and gas molecules, as well as provide a stable large-area reaction interface. On the other hand, the scaffold material can catalyze the cathodic charge–discharge reaction. And it even be used in unaqueous batteries to govern the development morphology of discharge products and provide adequate storage space for them. The logical design concept of NMS scaffolds is a crucial technique to improve MABs performance. However, due to the intricacy of the reaction variables, further investigation into the mechanism of NMS scaffold activity in air cathodes is required. In-depth analysis of the reaction process in combination with multiple characterization techniques is also necessary. Nonetheless, there are still challenges for the ideal design of NMS scaffolds applied to MABs. The following issues need to be considered from the perspectives of materials science and engineering: (1) Due to the complexity of the reaction variables at the air electrodes, the effects of the structural parameters on the gas, liquid, and internal charge transport need to be investigated in depth. Factors such as pore size, arrangement, number of active sites, and volume of mass-transfer space should be analyzed comprehensively to design the optimal structural parameters. (2) The precise preparation of NMS scaffolds is another major challenge to realizing their structural advantages. The use of high-precision preparation process should also consider the control of manufacturing cost. (3) In terms of material selection, in addition to the consideration of the conductivity of the scaffolds and their stability in electrolyte and air, the catalytic effect on the redox reaction can make the performance of the scaffolds go further.

## Summary and Perspectives

This review provides a comprehensive review of the advances in 3D NMS scaffolds for applications in EES over the past decade, from a structural perspective. As shown in Tables, NMS scaffolds, active materials, 3D NMS scaffolds, electrolytes, and electrochemical properties for SCs, AIBs, anodes of metal batteries, and cathodes of MABs are comprehensively summarized. Given the present progress, 3D NMS scaffolds with a continuous porous structure, sufficient voids, a large specific surface area, excellent mechanical properties, and a tunable structure have been shown to be an effective avenue to enhance the EES capacity. As for the design of NMS scaffolds, what we should always keep in mind is that high energy and power densities with a long cycle life are always the relentless pursuits of EES devices. In this perspective, it is obvious that the design principles of the 3D NMS scaffolds are complementary to the primary application mechanism. The main goal is to minimize the proportion of inactive components while maintaining or increasing the EES capacity per unit of active material. Another impressive feature of NMS scaffolds is the flexible design ability, which makes it possible to prepare predictable 3D electrodes according to the requirements of different applications, which can effectively improve the performance of the devices through rational structural design. This has, in turn, enabled further uncovering of the electrochemical behaviors at a more fundamental level due to the structure–function relationship for electrochemical reactions when combined with simulations.

**Fig. 14:**
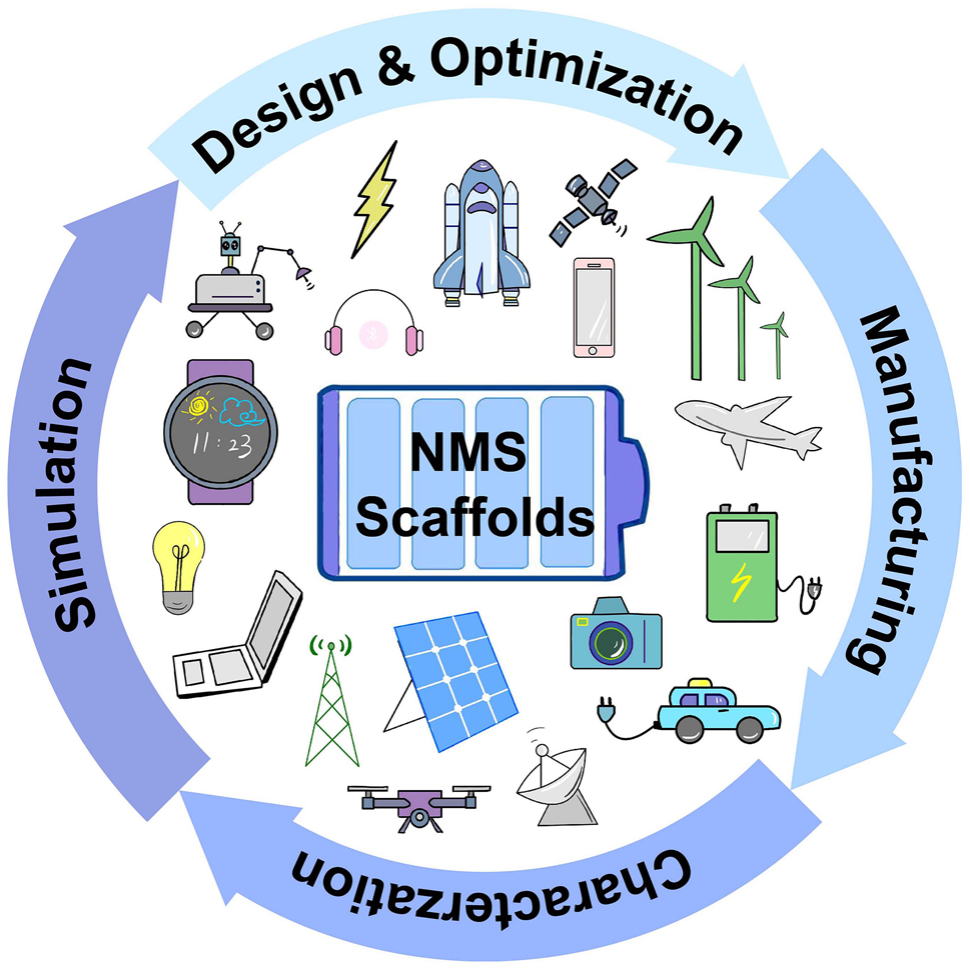
Development overview of NMS scaffolds through integration of advanced design, manufacturing, characterization, and simulation technologies

It is worth noting that despite a surge of new exciting NMS scaffolds tailored and constructed by fast developing nanotechnology and nano- and micro-fabrication techniques, the relevant research is still in its infancy. It is imperative to emphasize that some potential challenges should be considered in order to foster strengths and circumvent the weaknesses of NMS scaffolds toward further performance improvement in EES. First, enlarged surface area of electrochemical electrodes is probably accompanying with side reactions and the formation of insulating layers, which can be restrained via optimal design of NMS scaffold architectures and electrolytes at some extent. Furthermore, in order to prevent severed physical interfacing and attachment with other components after several cycles, rational integration of NMS scaffolds from design to assembly should be required. Another issue should pay attention to is the filiation of electrolyte especially for nano-structured scaffolds. Electrolyte penetration has had a severely impact on fully utilization of active materials in electrochemical reactions and transport kinetics of ions. In this fundamental viewpoint, rational design of NMS scaffolds with sufficient empty voids based on optimized volume ratio is essential for developing advanced NMS scaffolds for EES electrodes in practical applications. In fact, for solid-state electrolyte, there still remains challenging to realize fully electrolyte penetration in electrodes based on NMS scaffolds, which may be achieved via ALD technique in the near future. To this end, successfully bringing 3D NMS devices based on scaffolds from the laboratory to the market is still a daunting task, and much work remains to be done. Future works can be dedicated to the following aspects to realize the blueprint for the large-scale production of high-performance, stable EES devices (shown in Fig. 14). These would be discussed in the following:

(i) Design and optimization of electrode structures. Because of the design ability of 3D NMS scaffolds, it is possible to prepare 3D NMS scaffold electrodes with predictable structures precisely, enabling the customization of 3D structured electrodes in an application-led manner. Since the structure of 3D interconnected porous NMS-structured scaffolds has a great impact on the energy storage performance of the devices, it is important to have a deep understanding of the relationship between structure and electrochemical properties, such as specific surface area, porosity, and pore size distribution, and between structure and mechanical properties [190]. The ability to prepare precisely defined 3D NMS scaffolds, and thus precisely defined electrodes and devices, is of great significance, both in terms of performance enhancement and understanding the relationship between the relevant structure and electrochemical properties in combination with simulation techniques. This, in turn, will help researchers design and prepare more rational EES devices with better performance [191]. In addition, it is critical to control the pore size. For example, in SCs, if the pore size of the electrode is too small, the electrolyte ions cannot enter the entire electrode surface, which directly leads to a decrease in capacitance and an increase in resistance, affecting the overall performance of the device. However, when the pore size of the electrode is too large, the electrolyte ions may diffuse too fast in the electrode, leading to a decrease in capacitance and energy density as well as destruction of the electrode surface. To this end, designing and fabricating electrolyte more suitable for NMS scaffolds via advanced manufacturing techniques such as solid electrolyte in a conformal manner also are a practical and necessary aspect for practical applications [192].
(ii) Manufacturing technology. Although 3D NMS scaffolds have a great influence on the electrochemical performance of electrodes and devices, some applications require relatively large-size pores for electrodes, which are more in need of a wide range of inexpensive scaffold preparation processes. As for the miniature EES devices, the size of which is extremely limited, the distribution of the empty voids and the control of the pore size are becoming important. Hence, the realization of control for pore size and other parameters of 3D NMS scaffolds would be endowed by advanced preparation process technology, such as holographic lithography technology, holographic 3D printing, and so on. These technologies can directly prepare structures by inputting manufacturing parameters. However, their accuracy needs to be further improved at present. Accordingly, active material preparation processes compatible with 3D NMS scaffolds need to be carefully considered. For example, electrochemical deposition, CVD, ALD, and atomic layer etching have great potential for conformal thin-film-based active material preparation techniques.
(iii) Advanced characterization techniques. Advanced characterization technology methods are important for understanding the evolution of the morphology and structure of electrodes based on different 3D NMS scaffolds during electrochemical processes. For example, the study of the visualization of the anodic process in metal batteries is crucial for understanding the evolutionary mechanism of the growth process of metal dendrites. Among them, in-situ characterization techniques such as in-situ Raman spectroscopy, in-situ projection electron microscopy, in-situ nuclear magnetic resonance, and other characterization techniques are expected to further assist the research and development of high-performance EES electrodes.
(iv) Advanced simulation and analysis techniques. Simulation techniques and experiments are always in a complementary relationship. The design ability of the electrodes is realized due to the design ability of the 3D NMS scaffolds. Advanced theoretical simulation techniques, through modeling, can simulate the effect of the designed electrode structure on the electric field distribution and the electrolyte ion concentration gradient distribution, effectively predicting the rationality of the designed electrode [55–58]. For example, COMSOL is a powerful tool for simulating the growth of metal dendrites at the anode of metal batteries and the mass transfer effects at the three-phase interface of air electrodes, while DFT calculations can help researchers better understand the chemical reaction mechanisms such as the insertion–extraction process of active materials in batteries. When combined with advanced characterization and simulation analysis techniques, it is expected that the most suitable electrode structure design can be screened through extensive data analysis and mechanistic learning.

It is important to note that the study of 3D NMS scaffolds involves research areas such as chemistry, materials science, physics, and structural mechanics, which are interdisciplinary directions. At the same time, the preparation and integration of devices also involve engineering directions. By making 3D scaffolds the focus, it is aimed at pointing out the potential synergies between different disciplines and the road map for the EES field. In addition, the safety of the battery also needs to establish a reasonable evaluation system to avoid the occurrence of safety accidents [193–195]. With the increasing development of new energy sources, the treatment of waste batteries is also an emerging research direction [196]. We hope that this review can attract more researchers in related fields, especially in the field of power supply design engaged in energy harvesting, conversion, and storage, as well as in the fields of materials science, nanotechnology, and electronic device integration, to pay more attention to the 3D NMS scaffold strategy. This will accelerate the innovation and development of 3D NMS scaffolds in this field and promote EES devices with higher energy density, power density, and cycle life to meet the energy requirements of society and to follow the roadmap for sustainable energy development.
